# Immediate glucose signaling transmitted via the vagus nerve in gut–brain neural communication

**DOI:** 10.1016/j.isci.2025.112439

**Published:** 2025-05-05

**Authors:** Serika Yamada, Akiyo Natsubori, Kazuki Harada, Takashi Tsuboi, Hiromu Monai

**Affiliations:** 1 Department of Biology, Faculty of Science, Ochanomizu University, 2-1-1 Ohtsuka, Bunkyo-ku, Tokyo 112-8610, Japan; 2 Sleep Disorders Project, Tokyo Metropolitan Institute of Medical Science, 2-1-6 Kamikitazawa, Setagaya-ku, Tokyo 156-8506, Japan; 3 Department of Life Sciences, Graduate School of Arts and Sciences, The University of Tokyo, 3-8-1 Komaba, Meguro-ku, Tokyo 153-8902, Japan

**Keywords:** Molecular biology, Neuroscience, Microbiology

## Abstract

Sucrose consumption is influenced by certain gut–brain signaling mechanisms. One possible pathway could be the interaction between the vagus nerve and the central nervous system, mediated by neuropod cells forming synaptic connections with vagus nerves, which immediately activate the central dopaminergic pathways. In this study, we demonstrated that intestinal glucose administration activates the frontal cortex via the vagus nerve and central dopamine signaling. The immediate activation of both the vagus nerve and the frontal cortex was mediated by the sodium–glucose cotransporter 1 (SGLT1). Furthermore, Ca^2+^ signal activation in both astrocytes and neurons in the frontal cortex was mediated by D2 and D1 receptors, respectively. Finally, we showed that psychological stress, which causes a sucrose preference reduction, significantly diminished the activation levels of both the vagus nerve and the frontal cortex. These findings highlight the role of a comprehensive gut–brain network via vagus nerves in modulating sucrose preference.

## Highlights

• Gut glucose sensing via SGLT1 rapidly activates frontal cortex astrocytes and neurons
• Cortical astrocyte activation after gut glucose sensing robustly occurs also by D2DR
• Cortical neurons activation after gut glucose sensing robustly occurs so also by D1DR
• Frontal cortex activation after gut glucose sensing is absent in mice under CMRS

## Graphical abstract

**Figure:**
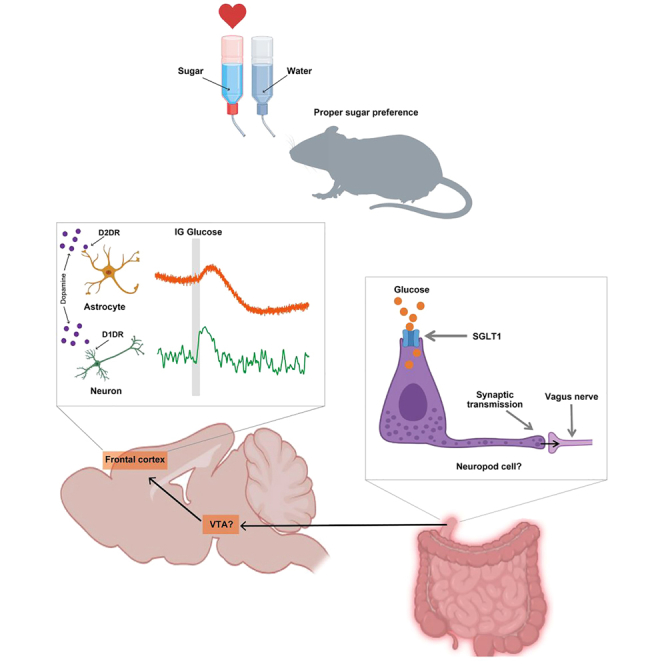

## Introduction

Glucose is one of the primary sources of energy in animals. In recent years, there has been increasing academic interest in the regulation of sugar (glucose or sucrose) consumption after sugar intake by signals transmitted via the vagus nerve, which afferently projects from the intestine to the brain.^1^^,^^2^^,^^3^^,^^4^^,^^5^^,^^6^ The central regulation of sugar intake via the afferent vagus nerve is conducted by complex pathways involving both inhibitory and stimulatory mechanisms; this variety of mechanisms is attributed to differences in projection targets corresponding to vagus nerve cell types.^7^^,^^8^^,^^9^^,^^10^ Gastrointestinal hormones (e.g., GLP-1) are representative compounds that inhibit feeding via a pathway mediated by vagus nerves. These hormones, as well as mechanosensing in gut, ultimately regulate feeding volume by regulating NPY/AgRP neurons and POMC/MSH neurons in the arcuate nucleus and paraventricular nucleus.^7^^,^^11^^,^^12^^,^^13^^,^^14^^,^^15^^,^^16^^,^^17^ While mechanisms of feeding inhibition operate via vagus nerves and involve the hypothalamus, the promotion of feeding through vagus nerves involves the interaction of the vagus nerve with the dopaminergic neurons' projection regions. Both pathways are crucial for maintaining sucrose preference.^18^^,^^19^^,^^20^^,^^21^ Recent findings have revealed that the activation of vagus nerves following glucose intake stimulates both pathways. Additionally, it has been suggested that the activation of the right vagus nerve primarily activates the nigrostriatal dopaminergic pathway, whereas the activation of the left vagus nerve predominantly activates the ventral tegmental area (VTA), which is the region in which the mesocortical and mesolimbic dopaminergic pathways originate.^22^^,^^23^^,^^24^^,^^25^^,^^26^^,^^27^

Gastrointestinal hormones contribute to both the inhibition and promotion of feeding via vagus nerves. Various gastrointestinal hormones are secreted minutes after glucose uptake in the intestine, with each hormone activating either the left or right vagus nerve. Consequently, several minutes after glucose reaches the intestine, the activation of both the nigrostriatal and mesolimbic pathway leads to dopamine release in the dorsal and ventral striatum, respectively, which promotes feeding.^23^^,^^28^ As mentioned above, this dopamine release induced by digestive hormones occurs minutes after glucose uptake in the intestine. On the other hand, recent observations have suggested that dopaminergic neurons in the VTA, originating from the neuropod cells followed by the left vagus nerve, are activated within seconds of administering sucrose into the intestine.^22^ Neuropod cells are a subtype of enteroendocrine cells, and it has been revealed that they can activate vagus nerves under intragastric (IG) sugar injection within 1 s by discharging glutamate as a neurotransmitter. These cells are predominantly situated in the duodenum and assimilate glucose through sodium–glucose co-transporter 1 (SGLT1). Signals derived from neuropod cells have been shown to contribute to the sucrose preference in mice.^29^^,^^30^^,^^31^^,^^32^^,^^33^^,^^34^ Despite these results, it had not yet been investigated where the activation of dopamine neurons in the VTA, presumed to originate from neuropod cells, subsequently leads to activation.

The frontal cortex is one of the principal projection targets of VTA dopaminergic neurons. This dopaminergic mesocortical pathway is activated by reward responses and plays a role in prompting appropriate behavior in response to rewarding stimuli.^18^^,^^35^^,^^36^^,^^37^^,^^38^^,^^39^^,^^40^^,^^41^ While the projection targets of VTA dopaminergic neurons encompass both the ventral striatum and the frontal cortex, a significant temporal divergence is observed; dopamine release in the ventral striatum occurs several minutes after intestinal glucose injection, whereas VTA dopaminergic neurons are activated promptly within seconds. This disparity suggests that the immediate activation of VTA dopaminergic neurons following intestinal glucose injection might involve the frontal cortex through a pathway distinct from that associated with the striatum. However, it remains unclear if neurons or astrocytes that are in the frontal cortex cells are immediately activated by the dopaminergic signals that are predicted to originate from neuropod cells.

These complex regulations described above result in a sucrose preference in a two-bottle choice test (sugar solution vs. water) in healthy mice. However, it is known that mice subjected to chronic psychological stress exhibit a marked decline in sucrose preference because of a decreased motivational drive for sucrose consumption and a reduced perception of pleasure after sucrose intake.^42^^,^^43^^,^^44^^,^^45^ While chronic psychological stress is implicated in dysregulating the brain’s reward system, recent reports have also underscored its influence on vagus nerve functionality.^46^^,^^47^^,^^48^^,^^49^^,^^50^^,^^51^^,^^52^^,^^53^ Given the role of vagus nerve–mediated dopaminergic neuron activation in motivation and pleasure, it is postulated that signals that are predicted to originate from neuropod cells may contribute to the attenuated sucrose preference observed after stress, although this hypothesis remains to be rigorously examined.

Building on existing literature, this study examined the immediate activation of the frontal cortex under direct intragastric (IG) glucose injection. Transcranial cortex-wide Ca^2+^ imaging and electrophysiological vagus nerve recordings were performed; then the signal was investigated in detail through fiber photometric recordings and two-photon Ca^2+^ imaging. Furthermore, this study evaluated potential modifications to neuropod cell–derived signaling in mice subjected to chronic mild restraint stress (CMRS), possibly derived from neuropod cells.

## Results

### Intragastric glucose injection immediately activates the vagus nerve, and it is inhibited by blocking sodium–glucose cotransporter 1

**Figure 1:**
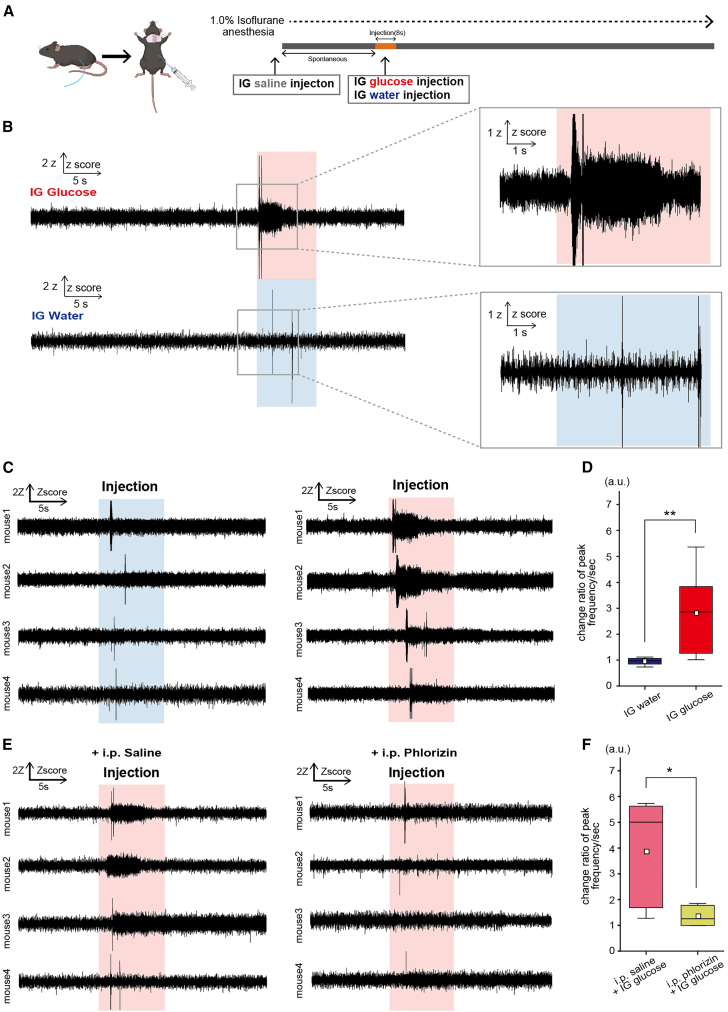
Vagal nerve response dynamics to IG glucose and water injections (A) Sequential representation of the vagus nerve recording procedure during IG injection. (B) Illustration of 50-Hz high-pass filtered vagus nerve activities under IG glucose (upper) and water (lower) injections. The injection periods are highlighted in light red and light blue, respectively. (C) Graph depicts the normalized frequencies of peaks per second in activities of individual mice receiving IG glucose (upper) or water (lower) injection. Normalization was based on the average baseline in the spontaneous state (30 s prior to IG injection). (D) Comparative analysis of changes in the frequencies of peaks per second in vagus nerve activityies in response to IG water (blue) or IG glucose (red) injection. The initial 30 s prior to IG injection are defined as the pre-stimulation phase, and the 2–4 s after IG injection are defined as the post-stimulation phase. ∗∗*p* < 0.01 (IG glucose, *n* = 9 mice; IG water, *n* = 8 mice; two-sample t-test). (E) Graphs illustrat the normalized frequencies of peaks per second in vagus nerve activities of individual mice receiving IG glucose injection following intraperitoneal (i.p.) phlorizin injection (upper data), or receiving IG glucose injection following i.p. saline injection (lower data). Normalization was performed on the basis of the average baseline in the spontaneous state (30 s prior to IG injection). (F) Comparative analysis of changes in the frequencies of peaks per second in vagus nerve activities in response to IG glucose injection after i.p. phlorizin injection (pink), or in response to IG glucose injection after i.p. saline injection (yellow). The initial 30 s prior to IG injection are designated as the pre-stimulation phase, and the 2–4 s after IG injection are defined as the post-stimulation phase. ∗∗*p* < 0.05 (IG glucose injection after i.p. saline injection, *n* = 5 mice; IG glucose injection after i.p. phlorizin injection, *n* = 6 mice; two-sample t-test). In each boxplot, the central box shows the average (mean) value, while the horizontal line within the box represents the median. Boxplot area represents the interquartile range (IQR). The upper and lower error bar in each boxplot represent the maximum and minimum value of each data excluding outliers. Outliers were defined as data points that fall outside the range of 1.5 times the IQR and were represented as black points.

To investigate the immediate response of the left vagus nerve to direct glucose delivery into the duodenum by using our experimental method, a 10% glucose solution was infused into the proximal duodenum of mice under anesthesia with 0.8–1.0% isoflurane. A pre-attached catheter was used to inject the glucose solution over 8 s (Figure 1A, see STAR Methods). IG glucose injection resulted in a notable activation of the left vagus nerve within 1 s (Figure 1B, top and C, top), in stark contrast to the absence of such activation after IG water injection (Figure 1B, bottom, C, bottom, and D, Water vs. Glucose: 0.96 ± 0.14 vs. 2.81 ± 1.56, t(15) = −3.36, *p* = 0.0043).

The swift propagation of signaling was postulated to involve neuropod cells, a specialized type of intestinal endocrine cell. Neuropod cells play a crucial role in glucose assimilation via SGLT1.^33^^,^^34^^,^^35^^,^^36^^,^^37^^,^^38^ IG glucose injection with the pretreatment of SGLT inhibitor phlorizin did not induce any significant vagus nerve activation (Figure 1E, top, bottom, and 1F, Saline vs. Phlorizin: 2.20 ± 0.98 vs. 0.39 ± 0.16, t(9) = 2.77, *p* = 0.02).

Given that neuropod cells are presently the only identified cells that induce immediate vagus nerve activations (within 1 s of glucose absorption) specifically after intestinal glucose absorption,^22^^,^^34^^,^^36^ the immediate vagus nerve response within 1 s only after IG glucose, not after IG water, injection implicates a mechanism originating from these cells. Additionally, the fact that the vagus nerve response was blocked by an SGLT1 inhibitor also indicates the involvement of neuropod cells.

### The frontal cortex undergoes immediate activation after intragastric glucose injection

**Figure 2:**
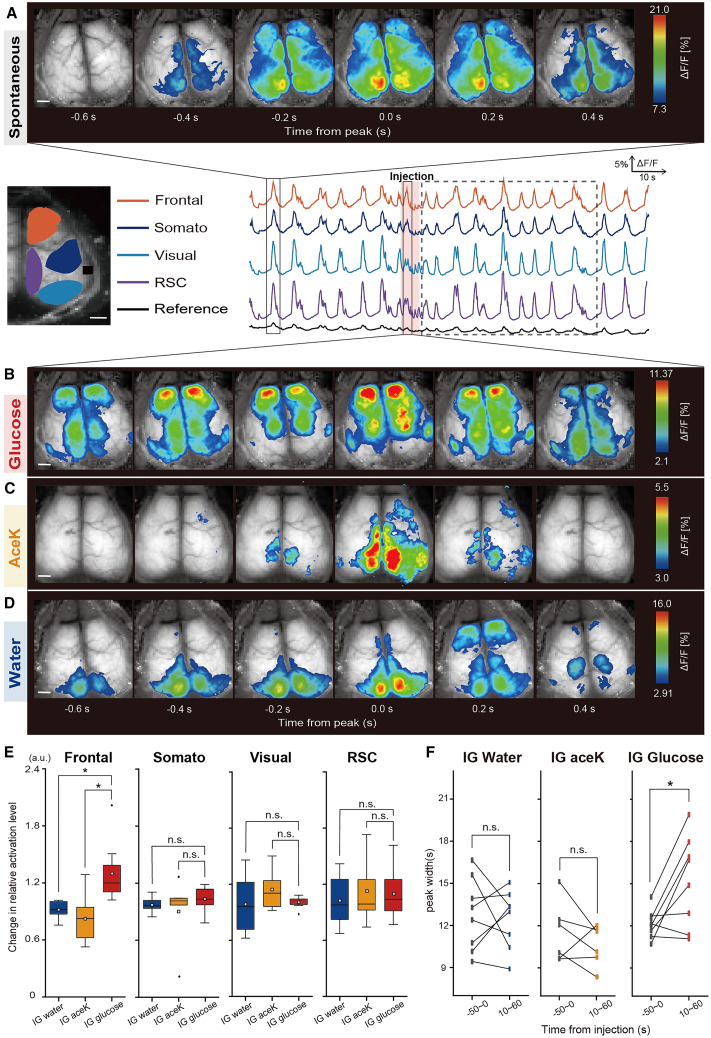
Differential cortical Ca^2+^ responses to IG glucose and water injection (A) Illustration of representative fluorescence intensity (ΔF/F) traces in G7NG817 mice for each cortical region (Frontal: orange, Somato: blue, Visual: light blue, RSC: purple, Reference: gray). The upper panel depicts spontaneous cortical Ca^2+^ activity patterns. The middle and lower panels depict cortical activity patterns after IG glucose or IG water injection, respectively. The analysis targets the earliest Ca^2+^ wave appearing within 3–8 s after the start of the injection. The pseudocolor representation employs the peak of the Ca^2+^ transient as the maximum value −1 SD, and the mean +1 SD as the minimum value. (B) Representative cortical activity patterns after IG glucose. The analysis targets the earliest Ca^2+^ wave appearing within 3–8 s after the start of the injection. Scale bar, 1mm. (C) Representative cortical activity patterns after IG AceK. The analysis targets the earliest Ca^2+^ wave appearing within 3–8 s after the start of the injection. Scale bar, 1mm. (D) Representative cortical activity patterns after IG water. The analysis targets the earliest Ca^2+^ wave appearing within 3–8 s after the start of the injection. Scale bar, 1mm. (E) Comparison of Ca^2+^ activation levels after IG glucose, AceK, or water injection in each cortical region. The data show the earliest peak value between 3 and 8 s after the start of the injection relative to the average of the peak value during the 50 s prior to the injection. ∗*p* < 0.05 (IG water: *n* = 8 mice; IG AceK: *n* = 5 mice; IG glucose: *n* = 8 mice; one-way ANOVA followed by Tukey-Kramer method). (F) Within-subject comparison of the widths of individual Ca^2+^ waves before (−50 to 0 s from injection, sponta) and in the later phase after injection (10–60 s from injection, IG (late)) of each group. Box area surrounded by dotted gray line depicts the later phase after injection. ∗*p* < 0.05 (left: IG water, *n* = 8 mice; middle: IG AceK, *n* = 5 mice; right: IG glucose, *n* = 8 mice; two-sample repeated t-test). In each boxplot, the central box shows the average (mean) value, while the horizontal line within the box represents the median. Boxplot area represents the interquartile range (IQR). The upper and lower error bar in each boxplot represent the maximum and minimum value of each data excluding outliers. Outliers were defined as data points that fall outside the range of 1.5 times the IQR and were represented as black points.

Next, transcranial cortex-wide Ca^2+^ imaging was conducted to assess the effect of IG glucose injection on cortical activity (Figures 2A–2D). This technique employed G7NG817 transgenic mice, which are characterized by the expression of the Ca^2+^ sensor protein G-CaMP7 that is under the control of the GLT-1 promoter and is expressed in astrocytes and excitatory neurons.^54^^,^^55^ Under isoflurane anesthesia, cortical spontaneous Ca^2+^ activity occurred regularly (Figure 2A). This Ca^2+^ activity was synchronized across intracortical regions, with relatively high amplitudes in the medial occipital regions.^56^^,^^57^^,^^58^ By contrast, the frontal cortex exhibited marked activation immediately after the start of the IG glucose injection (Figure 2B, middle), but not after the start of the IG acesulfame K (Ace K) or IG water injection (Figures 2C and 2D).

Quantitative analysis revealed that the Ca^2+^ activation induced by IG glucose injection was significant only in the frontal region (Figure 2E, Frontal cortex (Frontal): F(2, 18) = 6.75, *p* = 0.0065, Water vs. Glucose, 0.92 ± 0.08 vs. 1.30 ± 0.30, *p* = 0.019; Water vs. Ace K, 0.92 ± 0.08 vs. 0.84 ± 0.30, *p* = 0.85; Glucose vs. Ace K, 1.30 ± 0.30 vs. 0.84 ± 0.30, *p* = 0.013; Somatosensory cortex (Somato): F(2, 18) = 0.63, *p* = 0.54, Water vs. Glucose, 0.97 ± 0.072 vs. 1.04 ± 0.12, *p* = 0.82, Water vs. Ace K, 0.97 ± 0.072 vs. 0.90 ± 0.40, *p* = 0.83; Glucose vs. Ace K, 1.04 ± 0.12 vs. 0.90 ± 0.40, *p* = 0.52; Visual cortex (Visual): F(2, 18) = 0.20, *p* = 0.82, Water vs. Glucose, 0.99 ± 0.28 vs. 1.00 ± 0.06, *p* = 0.88; Water vs. Ace K, 0.99 ± 0.28 vs. 1.38 ± 0.38, *p* = 0.83; Glucose vs. Ace K, 1.00 ± 0.06 vs. 1.38 ± 0.38, *p* = 0.98; and Retrosplenial cortex (RSC): F(2, 18) = 0.90, *p* = 0.42, Water vs. Glucose, 1.02 ± 0.25 vs. 1.10 ± 0.26, *p* = 0.99, Water vs. Ace K, 1.02 ± 0.25 vs. 1.15 ± 0.24, *p* = 0.44; Glucose vs. Ace K, 1.10 ± 0.26 vs. 1.15 ± 0.24, *p* = 0.50). Moreover, we observed increases in the width of Ca^2+^ oscillations during the later phase (10–60 s from IG injection) compared to the spontaneous state (−50 to 0 s from IG injection), in the IG glucose injection group of mice (Figure 2F, Water: sponta. Vs. IG (late), 12.8 ± 0.92 vs. 12.5 ± 0.73, t(7) = 0.32, *p* = 0.76; Ace K: sponta. Vs. IG (late), 11.5 ± 0.88 vs. 10.6 ± 0.58, t(5) = 1.11, *p* = 0.32; Glucose: sponta. Vs. IG (late), 12.1 ± 0.36 vs. 15.2 ± 1.13, t(7) = −3.0, *p* = 0.019). These results indicate that IG glucose injection immediately induces frontal cortex activation, which is not caused by the entry of fluid into the intestines or by the osmolarity of the solution.

### Immediate activation of the frontal cortex after intragastric glucose injection involves the sodium–glucose cotransporter 1 and dopamine

**Figure 3:**
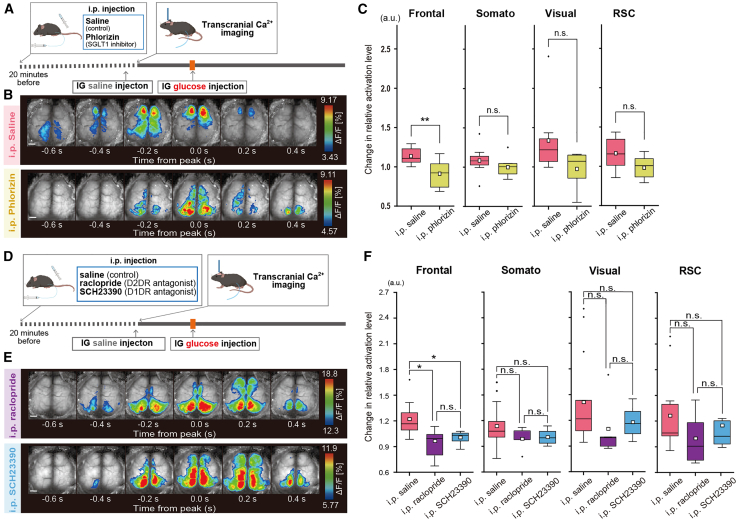
Influence of SGLT1 and dopaminergic antagonists on cortical Ca^2+^ activation after IG glucose injection (A) Schema of transcranial cortex-wide Ca^2+^ imaging under IG glucose injection with intraperitoneally administered phlorizin (SGLT1 antagonist) or saline (control). (B) Illustration of cortical activity patterns after IG glucose injection with saline (upper) or phlorizin (lower). The analysis targeted the earliest Ca^2+^ wave appearing within 3–8 s after injection. The pseudocolor representation shows the peak of the Ca^2+^ transient as the maximum value −1 SD, and the mean +1 SD as the minimum value. Scale bar, 1mm. (C) Comparison of Ca^2+^ activation levels in each cortical region after IG glucose injection with saline or phlorizin pretreatment. The peak fluorescence intensity value of each brain region was compared against that of a reference region (auditory cortex), encompassing the 50 s prior to injection and the subsequent post-injection period. Post-injection values were normalized by dividing them by the pre-injection average and compared between different treatment groups. ∗∗*p* < 0.01 (saline, *n* = 8 mice; phlorizin; *n* = 7 mice; two-sample t-test). (D) Schema of transcranial cortex-wide Ca^2+^ imaging under IG glucose injection with the i.p. administration of saline (control), raclopride (D2DR antagonist), or SCH23390 (D1DR antagonist). (E) Illustration of cortical activity patterns after IG glucose injection with the i.p. administration of raclopride (upper) or SCH23390 (lower). The analysis focused on the earliest Ca^2+^ wave occurring within 3–8 s after injection. The pseudocolor representation shows the peak of the Ca^2+^ transient as the maximum value – 1 SD, and the mean +1 SD as the minimum value. Scale bar, 1mm. (F) Comparative analysis of activation levels in each cortical region of mice after IG glucose injection with saline (left), raclopride (middle), or SCH23390 (right) intraperitoneal pretreatment. The peak fluorescence intensity value of each brain region was compared against that of a reference region (auditory cortex), encompassing the 50 s prior to injection and the subsequent post-injection period. Post-injection values were normalized by dividing them by the pre-injection average and compared across different treatment groups. ∗*p* < 0.05 (saline: *n* = 7 mice; raclopride: *n* = 7 mice; SCH23390: *n* = 8 mice; one-way ANOVA followed by Tukey-Kramer method). In each boxplot, the central box shows the average (mean) value, while the horizontal line within the box represents the median. Boxplot area represents the interquartile range (IQR). The upper and lower error bar in each boxplot represent the maximum and minimum value of each data excluding outliers. Outliers were defined as data points that fall outside the range of 1.5 times the IQR and were represented as black points.

In Figure 1, we observed a significant reduction in the immediate activation of the left vagus nerve under IG glucose injection in the presence of phlorizin, an inhibitor against SGLT1 crucial for glucose uptake by neuropod cells. On the basis of these results, similar transcranial cortex-wide Ca^2+^ imaging was performed in the presence of phlorizin to determine the contribution of SGLT1 to frontal cortex activation following IG glucose injection (Figure 3A).

In the presence of phlorizin, no activation of the frontal cortex was observed after IG glucose injection (Figures 3B and 3C, Frontal: Saline vs. Phlorizin, 1.01 ± 0.20 vs. 0.91 ± 0.15, t(14) = −3.06, *p* = 0.0080; Somato: Saline vs. Phlorizin, 1.08 ± 0.17 vs. 1.00 ± 0.12, t(14) = −1.45, *p* = 0.33; Visual: Saline vs. Phlorizin, 1.34 ± 0.42 vs. 0.97 ± 0.20, t(14) = −2.032, *p* = 0.078; and RSC: Saline vs. Phlorizin, 1.16 ± 0.19 vs. 1.00 ± 0.13, t(14) = −1.84, *p* = 0.095). Moreover, to verify that the characteristic responses observed after IG glucose injection are mediated by vagus nerves, similar transcranial cortex-wide Ca^2+^ imaging was conducted in mice that underwent either vagotomy or a sham operation. Compared with sham operation, left vagotomy eliminated the immediate activation of the frontal cortex and the peak width increase in the later phase (Figures S1A and S1B). These results revealed that the immediate activation of the left vagus nerve, as well as the activation of the frontal cortex, was mediated by glucose uptake via SGLT1 after IG glucose injection.

Previous reports have suggested that the centrifugal vagus nerves, which form synapses with neuropod cells, project to the nucleus of the solitary tract and immediately activate VTA dopaminergic neurons.^22^ To investigate the potential involvement of dopamine in the frontal cortex activation induced by IG glucose injection, as seen in Figure 2, similar transcranial cortex-wide Ca^2+^ imaging was performed in the presence of the D1 dopamine receptor (D1DR) antagonist SCH23390 or the D2 dopamine receptor (D2DR) antagonist raclopride. Compared with control, both SCH23390 and raclopride eliminated the activation of the frontal cortex after IG glucose injection (Figures 3D–3F, Frontal: F(2, 19) = 5.51, *p* = 0.012, Saline vs. Raclopride, 1.26 ± 0.25 vs. 0.94 ± 0.22, *p* = 0.014; Raclopride vs. SCH23390, 0.94 ± 0.22 vs. 1.01 ± 0.07, *p* = 0.75; Saline vs. SCH23390, 1.26 ± 0.25 vs. 1.01 ± 0.07, *p* = 0.05; Somato: F(2, 19) = 2.05, *p* = 0.16, Saline vs. Raclopride, 1.18 ± 0.29 vs. 0.96 ± 0.21, *p* = 0.16; Raclopride vs. SCH23390, 0.96 ± 0.21 vs. 1.01 ± 0.09, *p* = 0.90; Saline vs. SCH23390, 1.18 ± 0.29 vs. 1.01 ± 0.09, *p* = 0.29; Visual: F(2, 19) = 0.86, *p* = 0.14, Saline vs. Raclopride, 1.45 ± 0.58 vs. 1.06 ± 0.18, *p* = 0.13; Raclopride vs. SCH23390, 1.06 ± 0.18 vs. 1.18 ± 0.17, *p* = 0.81; Saline vs. SCH23390, 1.45 ± 0.58 vs. 1.18 ± 0.17, *p* = 0.32; and RSC: F(2, 19) = 2.19, *p* = 0.14, Saline vs. Raclopride, 1.38 ± 0.53 vs. 1.15 ± 0.29, *p* = 0.47; Raclopride vs. SCH23390, 1.15 ± 0.29 vs. 1.18 ± 0.24, *p* = 0.99; Saline vs. SCH23390, 1.38 ± 0.53 vs. 1.18 ± 0.14, *p* = 0.54).

### Immediate activation of the frontal cortex after intragastric glucose injection encompasses both neurons and astrocytes

**Figure 4:**
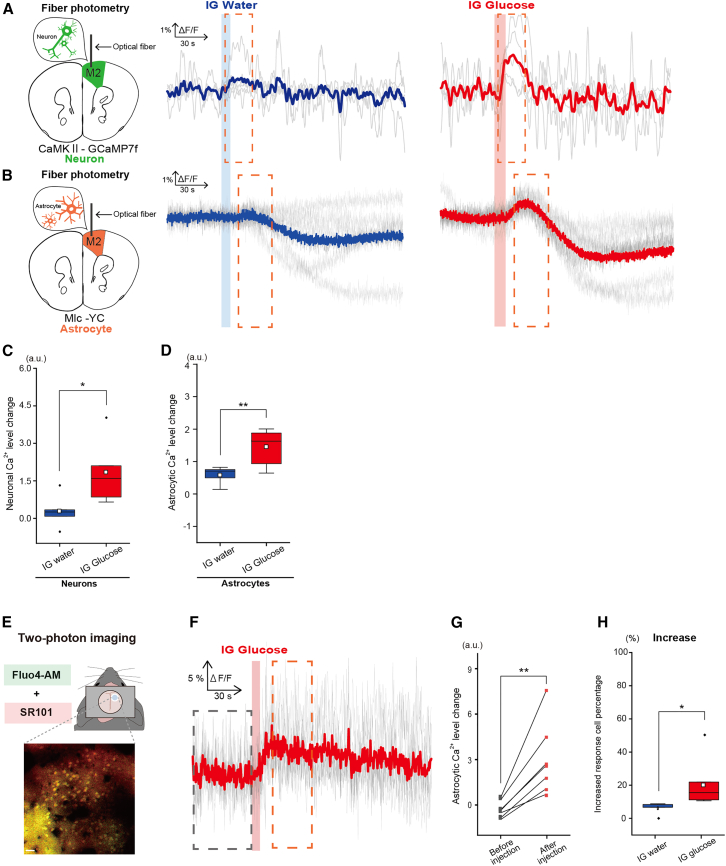
Neuronal and astrocytic Ca^2+^ dynamics in response to IG glucose or water injection (A) Fiber photometric traces of averaged CaMKII-GCaMP7f (Neurons) signal responses to IG water injection (left) or IG glucose injection (right), with the light gray line depicting individual mouse traces. (IG glucose, *n* = 5 mice; IG water, *n* = 5 mice). (B) Fiber photometric traces of averaged Mlc-YC (Astrocytes) signal responses to IG water injection (left) or IG glucose injection (right), with the light gray line representing individual mouse traces. (IG glucose; *n* = 7 mice, IG water; *n* = 5 mice). (C) Comparison of Ca^2+^ activation levels after IG water (left) or glucose (right) injection in CaMKII-GCaMP7f (Neurons) mice. The analysis window corresponds to the area within the dotted line in (A). ∗*p* < 0.05 (IG glucose, *n* = 5 mice; IG water, *n* = 5 mice; two-sample t-test). (D) Comparison of Ca^2+^ activation levels (early phase) after IG water (left) or glucose (right) injection in Mlc-YC (Astrocytes) mice. The analysis window corresponds to the area within the dotted line in (B). ∗∗*p* < 0.01 (IG glucose, *n* = 7 mice; IG water, *n* = 5 mice; two-sample t-test). (E) Representative two-photon Ca^2+^ imaging of layer 2 in the frontal cortex, with cells labeled with Fluo4-AM (green; Ca^2+^ indicator) and SR101 (red; astrocytes). Scale bar, 10μm. (F) ΔF/F traces in the cell population judged to exhibit an increase in fluorescence intensity after IG glucose injection (gray line), alongside the corresponding average data (bold red line). (25 cells from *n* = 7 mice). (G) Comparison within each individual of the average value of astrocytic ΔF/F traces in response to IG glucose injection (15–38 s after injection; within the red dotted line in (F)) against the spontaneous state (50 s prior to injection; within the gray dotted line in (F)). ∗∗*p* < 0.01 (IG glucose, *n* = 7 mice; two-sample repeated t-test). (H) Comparison of the percentage of all SR101-positive cells exhibiting an increased Fluo4-AM signal. ∗*p* < 0.05 (IG glucose, *n* = 7 mice; IG water, *n* = 5 mice; welch’s t-test). In each boxplot, the central box shows the average (mean) value, while the horizontal line within the box represents the median. Boxplot area represents the interquartile range (IQR). The upper and lower error bar in each boxplot represent the maximum and minimum value of each data excluding outliers. Outliers were defined as data points that fall outside the range of 1.5 times the IQR and were represented as black points.

The G7NG817 transgenic mice used in Figures 2 and 3 revealed Ca^2+^ signaling in both neurons and astrocytes, making it unclear which cell type is implicated in frontal cortex activation after IG glucose injection. To clarify this issue, cell type–specific Ca^2+^ recording was performed in the frontal cortex using mice injected with AAV9 CaMKⅡa-GCaMP7f (express excitatory neurons specific Ca^2+^ indicator) and Mlc1-tTAtetO-YC-Nano50 transgenic mice (expressing astrocytes specific Ca^2+^ indicator), accompanied by fiber photometry (Figures 4A, 4B, and S2). The analysis showed a significant augmentation in fluorescence signals within both neurons and astrocytes in the frontal cortex after IG glucose injection as opposed to water (Figures 4C and 4D, Neurons: Water vs. Glucose, 0.29 ± 0.67 vs. 1.85 ± 1.35, t(8) = −2.31, *p* = 0.049; Astrocytes: Water vs. Glucose, 0.48 ± 0.28 vs. 1.46 ± 0.51, t(10) = 3.46, *p* = 0.006).

While IG glucose injection elicited activation in both neurons and astrocytes within the frontal cortex, the activation patterns were distinct between the two cell types. Neurons displayed a transient surge in fluorescence signal that subsequently reverted to baseline, whereas in astrocytes, a transient increase was followed by a decrease below baseline. The reductions in fluorescence signal occurred roughly 40 s after injection with glucose or water, with no significant difference between the two (Figures S3A and S3B).

The fluorescence signals captured via fiber photometry reflect Ca^2+^ activity at the population level among cell groups expressing Ca^2+^-sensitive fluorescent proteins within the analyzed brain region. The results of fiber photometry revealed that while neurons exhibit a simple Ca^2+^ response (increase or lack of increase), astrocytes demonstrate a complex response pattern in which the Ca^2+^ level decreases in the late phase after IG injection. Thus, two-photon Ca^2+^ imaging was performed to investigate whether this complex response was due to synchronous cellular activity triggered by a signal occurring in the late phase (Figure 4E). Fluo-4 was used as a two-photon Ca^2+^ indicator to simultaneously address the possibility that the complex signal fluctuations observed only in astrocytes could be attributed to differences in the Kd values of the GCaMP7f and YC probes. The findings revealed that the Ca^2+^ level of some cells labeled with sulforhodamine 101 (SR101), a marker of astrocytes, increased after IG glucose injection (Figures 4F and 4G, Before injection vs. After injection, −0.12 ± 0.22 vs. 3.04 ± 2.39, t(6) = −2.31, *p* = 0.01). After IG glucose injection, approximately 21% of cells showed an increase in fluorescence intensity, and this percentage was significantly greater than that observed after water injection. We determined that cells exhibiting fluorescence intensity surpassing the average +0.5 × standard deviation (SD) as increase in fluorescence intensity (Figure 4H, Water vs. Glucose, 6.01 ± 3.52 vs. 20.0 ± 13.4, t(10) = 2.19, *p* = 0.036). These results indicate the involvement of both neurons and astrocytes in the immediate activation of the frontal cortex after IG glucose injection.

### The activation of the frontal cortex after intragastric glucose injection is mediated by dopamine, with astrocytes and neurons responding through distinct receptors

**Figure 5:**
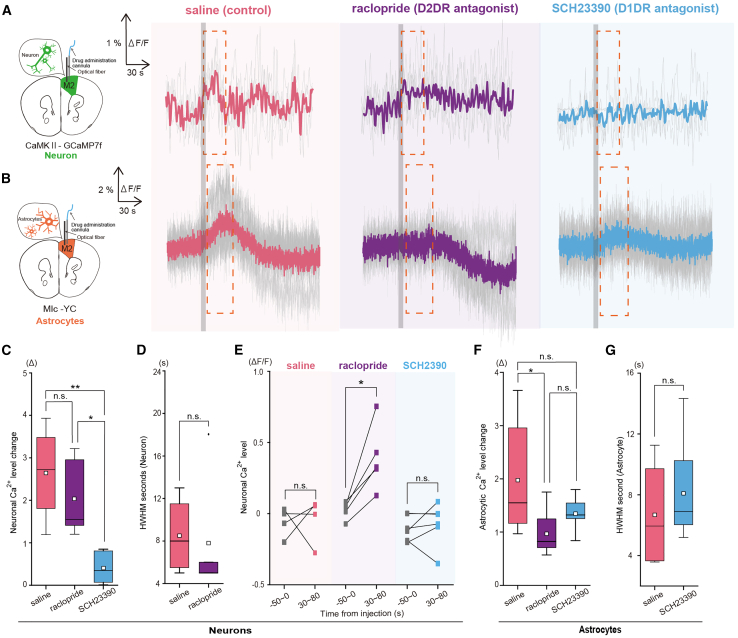
Ca^2+^ activity of neurons and astrocytes in response to IG glucose injection under the local inhibition of dopamine receptors (A) Fiber photometric traces of averaged CaMKII-GCaMP7f (Neurons) signal responses to IG glucose injection with saline (left), raclopride (middle), or SCH23390 (right) local pretreatment. The gray shaded area denotes the IG glucose injection period. (saline, *n* = 5 mice; raclopride, *n* = 6 mice; SCH23390, *n* = 5 mice). (B) Fiber photometric traces of averaged Mlc-YC (Astrocytes) signal responses to IG glucose injection with saline (left), raclopride (middle), or SCH23390 (right) local pretreatment. The gray shaded area indicates the IG glucose injection period. (saline, *n* = 5 mice; raclopride, *n* = 8 mice; SCH23390, *n* = 7 mice). (C) Comparison of Ca^2+^ activation levels after the IG glucose injection of CaMKII-GCaMP7f (Neurons) in mice pretreated with saline (left), raclopride (middle), or SCH23390 (right). †*p* < 0.1, ∗*p* < 0.05 (saline, *n* = 5 mice; raclopride, *n* = 6 mice; SCH23390, *n* = 5 mice; one-way ANOVA followed by Tukey–Kramer method). (D) Comparison of the HWHM of CaMKII-GCaMP7f (Neurons) signals after IG glucose injection with saline (pink) or raclopride (purple) pretreatment. (saline, *n* = 5 mice; raclopride, *n* = 6 mice; t-test). (E) Within-subject comparison of neuronal Ca^2+^ level between before (−50 to 0 s from injection) and later phase (30–80 s from injection) after IG glucose injection with saline (left), raclopride (middle), or SCH23390 (right) local pretreatment. ∗*p* < 0.05 (saline: *n* = 4 mice; raclopride: *n* = 5 mice; SCH23390: *n* = 6 mice; two-sample repeated t-test). (F) Comparison of Ca^2+^ activation (early-phase) levels after IG glucose injection in Mlc-YC (Astrocytes) mice locally pretreated with saline (left), raclopride (middle), or SCH23390 (right). ∗*p* < 0.05 (saline, *n* = 6 mice; raclopride, *n* = 7 mice; SCH23390, *n* = 7 mice; one-way ANOVA followed by Tukey–Kramer method). (G) Comparison of HWHM of Mlc-YC (Astrocytes) signals under IG glucose injection with saline (pink) or SCH23390 (light blue) local pretreatment. (saline, *n* = 6 mice; SCH23390, *n* = 7 mice; two-sample t-test). In each boxplot, the central box shows the average (mean) value, while the horizontal line within the box represents the median. Boxplot area represents the interquartile range (IQR). The upper and lower error bar in each boxplot represent the maximum and minimum value of each data excluding outliers. Outliers were defined as data points that fall outside the range of 1.5 times the IQR and were represented as black points.

Transcranial Ca^2+^ imaging revealed that dopamine signaling, mediated by D1DR and D2DR, played a role in the activation of the frontal cortex (Figures 3D–3F). Typically, frontal cortex neurons propagate reward-related signals via D1DR.^59^^,^^60^^,^^61^^,^^62^ Although the response of individual astrocytes to dopamine via each receptor has been thoroughly investigated, there has been minimal research into the detailed mechanism of astrocyte responses to dopamine in the frontal cortex under physiological conditions.^63^^,^^64^^,^^65^^,^^66^ To delineate the contributions of D1DR and D2DR to the activation of neurons and astrocytes after IG glucose injection, the effects of local D1DR and D2DR inhibition on CaMKII-GCamp7f and Mlc-YC signals were assessed using fiber photometry (Figure 5).

In neurons, the administration of a D1DR antagonist (SCH23390), but not control, CaMKII-GCamp7f signal, significantly reduced the increase in fluorescence intensity that occurred after IG glucose injection. However, a persistent elevation in fluorescence intensity was observed after the administration of both control and a D2DR antagonist (raclopride), with no significant differences in amplitude or activation duration between them (Figures 5A, 5C, and 5D, Amplitude: F(2, 13) = 10.10, *p* = 0.0027, Saline vs. Raclopride, 0.53 ± 0.11 vs. 0.41 ± 0.08, *p* = 0.37; Raclopride vs. SCH23390, 0.41 ± 0.08 vs. 0.08 ± 0.03, *p* = 0.018; Saline vs. SCH23390, 0.53 ± 0.11 vs. 0.08 ± 0.03, *p* = 0.004; Half-width at half maximum (HWHM): Saline vs. Raclopride, 8.5 ± 3.20 vs. 7.8 ± 5.11, t(7) = 0.21, *p* = 0.84).

In astrocytes, the increase in fluorescence intensity was significantly reduced by the administration of raclopride. Conversely, elevated fluorescence intensity after IG glucose injection was still observed after the administration of saline or SCH23390, with no differences in amplitude or HWHM between them (Figures 5B and 5E–5G, Amplitude: F(2, 17) = 3.78, *p* = 0.04, Saline vs. Raclopride, 1.86 ± 1.21 vs. 0.60 ± 0.70, *p* = 0.044; Raclopride vs. SCH23390, 0.60 ± 0.70 vs. 1.25 ± 0.55, *p* = 0.325; Saline vs. SCH23390, 1.86 ± 1.21 vs. 1.25 ± 0.55, *p* = 0.42; HWHM: Saline vs. SCH23390, 3.45 ± 1.54 vs. 4.11 ± 1.55, t(11) = −0.78, *p* = 0.95).

Taken together, these findings suggest that the activation of the frontal cortex after IG glucose injection could result from neuronal and astrocytic activation through D1DR- and D2DR-mediated dopaminergic signaling, respectively. Additionally, we confirmed that the intraperitoneal administrations of D1DR and D2DR antagonists had similar effects on the neuronal and astrocytic Ca^2+^ signals in the frontal cortex as the local administration (Figure S4), and the intraperitoneal administration of D2DR antagonist only decreases the acute sucrose preference (Figure S5). It should be noted that our experiment investigating the role of dopamine in sucrose preference is determined by multiple factors and does not solely examine vagal nerve-mediated responses. However, the findings suggest that astrocyte D2 receptor-mediated responses may contribute as one factor to acute sucrose preference through D2 receptors.

### Chronic mild restraint stress decreases the immediate activation of the vagus nerve and frontal cortex after intragastric glucose injection

**Figure 6:**
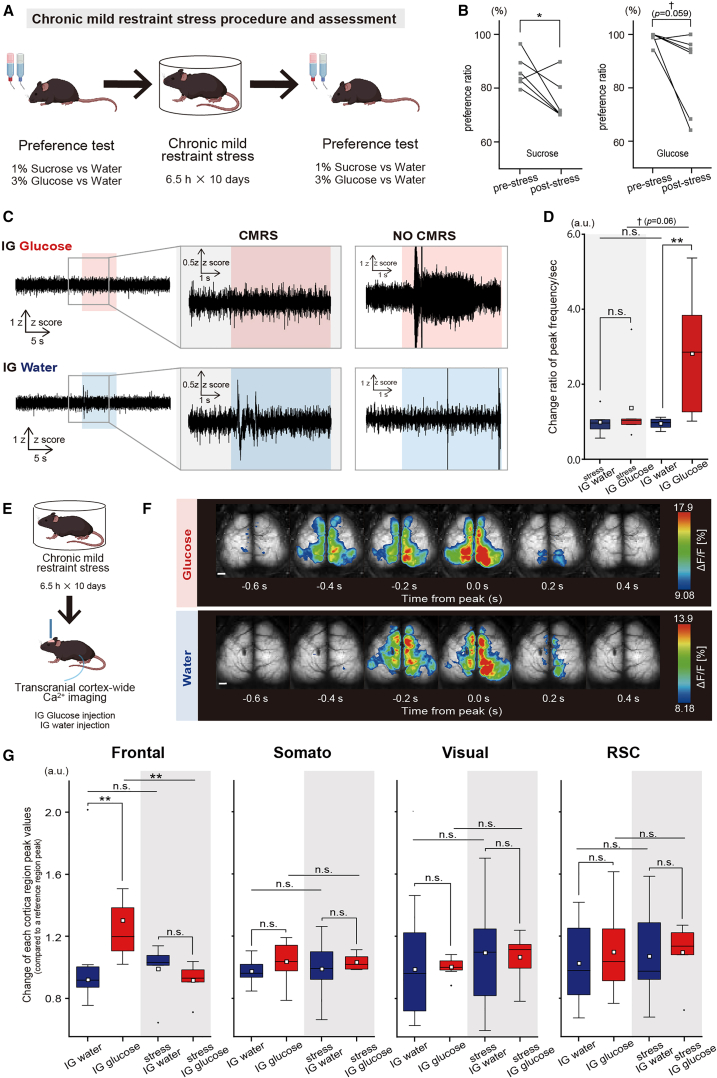
Impact of CMRS on vagus and cortical responses to IG glucose injection (A) Overview of CMRS methodology and assessment. (B) Changes in the preference ratio for 1% sucrose (left) or 3% glucose (right) before and after stress. ∗*p* < 0.05, †*p* < 0.1 (Sucrose preference test: *n* = 6 groups derived from 12 mice, two-sample paired t-test; Glucose preference test: *n* = 6 groups from 12 mice, Wilcoxon signed-rank sum test). (C) Illustration of normalized 50-Hz high-pass filtered vagal nerve activity after IG glucose (upper) or IG water (lower) injection in mice subjected to CMRS (dotted purple line) and without CMRS (dotted orange line). The injection period is highlighted in light red (IG glucose) or light blue (IG water). (CMRS; glucose, *n* = 6 mice; water, *n* = 5 mice). (D) Changes in the frequencies of peaks per second in vagus nerves before and after the IG injection of glucose (red) or water (blue). The data surrounded by a dotted purple line represent the group after CMRS, whereas those surrounded by a dotted orange line represent the group without CMRS. The initial 30 s before IG injection is considered the pre-stimulation period, while the 2–4 s after IG injection is considered the post-stimulation period. ∗∗*p* < 0.01, †*p* < 0.1 (Untreated IG water: *n* = 8 mice; Untreated IG glucose: *n* = 9 mice; Stressed IG water: *n* = 5 mice; Stressed IG glucose: *n* = 6 mice; one-way ANOVA followed by Tukey–Kramer method). (E) Schema of transcranial cortex-wide Ca^2+^ imaging after CMRS. (F) Depiction of cortical activity patterns after IG glucose (upper) or water (lower) injection in mice subjected to CMRS. The calcium wave targeted for analysis was the earliest wave appearing within 3–8 s after injection. The pseudocolor representation shows the peak of the Ca^2+^ transient as the maximum value −1 SD, and the mean +1 SD as the minimum value. Note that mice subjected to CMRS exhibited no activation of the frontal cortex after IG glucose injection. Scale bar, 1 mm. (G) Comparison of activation levels in each cortical region of post-stress mice after IG water (blue) or glucose (red) injection. The peak fluorescence intensity values in each brain region were compared against those of a reference region (auditory cortex) over a designated time window, including the 50 s before and after injection. Post-injection values were normalized by dividing them by the pre-injection average and compared between different treatment groups. The data on the white background represent the group without CMRS, whereas those on the gray background represent the group after CMRS exposure. ∗∗*p* < 0.01 (Untreated IG water: *n* = 8 mice; Untreated IG glucose: *n* = 8 mice; Stressed IG water: *n* = 7 mice; Stressed IG glucose: *n* = 6 mice; one-way ANOVA followed by Tukey–Kramer method). In each boxplot, the central box shows the average (mean) value, while the horizontal line within the box represents the median. Boxplot area represents the interquartile range (IQR). The upper and lower error bar in each boxplot represent the maximum and minimum value of each data excluding outliers. Outliers were defined as data points that fall outside the range of 1.5 times the IQR and were represented as black points.

Finally, to investigate whether the immediate signals following glucose uptake are involved in the decreased sucrose preference caused by chronic restraint stress, we examined the effects of mouse CMRS on the activation of the left vagus nerve and frontal cortex. A CMRS model was established by confining mice in plastic tubes for 6.5 h daily for 10 days, followed by sucrose and glucose preference tests before and after CMRS exposure (Figure 6A). The results demonstrated a significant decrease in both sucrose and glucose preference (Figure 6B, Sucrose preference test: Pre-stress vs. Post-stress, 0.87 ± 0.06 vs. 0.75 ± 0.08, t(5) = 2.56, *p* = 0.048; Glucose preference test: Pre-stress vs. Post-stress, 0.99 ± 0.02 vs. 0.86 ± 0.16, t(5) = 1.89, *p* = 0.059).

Subsequent measurements of vagus nerve activities after IG glucose or water injection in CMRS mice (Figure 6C) revealed that the activation observed after glucose injection in healthy mice was absent in stressed mice (Figure 6D, Untreated: F(2, 27) = 5.90, *p* = 0.0036, Water vs. Glucose, 0.96 ± 0.14 vs. 2.81 ± 1.56, *p* = 0.006; Stressed: Water vs. Glucose, 0.99 ± 0.36 vs. 1.34 ± 1.03, *p* = 0.93; Untreated water vs. Stressed water, 0.96 ± 0.14 vs. 0.99 ± 0.36, *p* = 0.99; Untreated glucose vs. Stressed glucose, 2.81 ± 1.56 vs. 1.34 ± 1.03, *p* = 0.06).

Given that CMRS mice exhibited reduced vagus nerve activations, it was expected that they would show a corresponding decrease in frontal cortex activation, which is typically noted in healthy mice. Transcranial cortex-wide Ca^2+^ imaging after IG glucose or water injection (Figures 6E and 6F) confirmed a significant reduction in frontal cortex activation after IG glucose injection in CMRS mice, with no significant amplitude difference relative to water injection (Figure 6G, Frontal: F(3, 26) = 6.1, *p* = 0.003, Untreated water vs. Untreated glucose, 0.92 ± 0.09 vs. 1.30 ± 0.32, *p* = 0.005; Stressed water vs. Stressed glucose, 0.99 ± 0.18 vs. 0.92 ± 0.11, *p* = 0.93; Untreated water vs. Stressed water, 0.92 ± 0.09 vs. 0.99 ± 0.18, *p* = 0.92; Untreated glucose vs. Stressed glucose, 1.30 ± 0.32 vs. 0.92 ± 0.11, *p* = 0.009; Somato: F(3, 26) = 0.46, *p* = 0.71, Untreated water vs. Untreated glucose, 0.97 ± 0.08 vs. 1.04 ± 0.13, *p* = 0.75; Stressed water vs. Stressed glucose, 0.99 ± 0.20 vs. 1.03 ± 0.05, *p* = 0.93, Untreated water vs. Stressed water, 0.97 ± 0.08 vs. 0.99 ± 0.20, *p* = 0.99; Untreated glucose vs. Stressed glucose, 1.04 ± 0.13 vs. 1.03 ± 0.05, *p* = 0.99; Visual: F(3, 26) = 0.12, *p* = 0.44, Untreated water vs. Untreated glucose, 0.99 ± 0.30 vs. 1.00 ± 0.06, *p* = 0.99; Stressed water vs. Stressed glucose, 1.09 ± 0.39 vs. 1.07 ± 0.16, *p* = 0.99; Untreated water vs. Stressed water, 0.99 ± 0.30 vs. 1.09 ± 0.39, *p* = 0.86; Untreated glucose vs. Stressed glucose, 1.00 ± 0.06 vs. 1.07 ± 0.16, *p* = 0.97; RSC: F(3, 26) = 2.20, *p* = 0.14, Untreated water vs. Untreated glucose, 1.02 ± 0.27 vs. 1.1 ± 0.27, *p* = 0.95; Stressed water vs. Stressed glucose, 1.07 ± 0.32 vs. 1.09 ± 0.20, *p* = 0.99; Untreated water vs. Stressed water, 1.02 ± 0.27 vs. 1.07 ± 0.32, *p* = 0.99; Untreated glucose vs. Stressed glucose, 1.1 ± 0.27 vs. 1.09 ± 0.20, *p* = 0.99). These results suggest that the immediate activation of the frontal cortex in response to IG glucose injection mediates the left vagus nerve and the central dopaminergic system.

## Discussion

This study showed that the immediate vagus nerve activation induced by direct duodenal glucose injection (IG glucose injection) was mediated by SGLT1 that have a role in transporting glucose within neuropod cells (Figure 1). Furthermore, this signal activated both astrocytes and neurons in the frontal cortex, as shown by transcranial cortex-wide Ca^2+^ imaging (Figures 2 and 3) and fiber photometry (Figures 4 and 5). Pharmacological evidence suggested the involvement of D2DR in astrocytic activation and of D1DR in neuronal activation in the frontal cortex (Figures 3, 5, and S5). As local administrations of D1DR or D2DR antagonists inhibited neuronal and astrocytic Ca^2+^ level upregulation respectively, the dopamine pathway activated by IG glucose injection is suggested to be a mesocortical pathway (Figure 5). Additionally, activation level of this immediate signals was altered at the stage of signal transmission to the vagus nerve in CMRS mice (Figure 6).

### The immediate activation of the frontal cortex following intestinal glucose administration would be attributed to the activation of the mesocortical dopaminergic pathway originating from neuroepithelial cells

Previous research has shown that sugar absorption in the gut triggers the activation of central dopaminergic pathways that can be broadly divided into the following: the nigrostriatal, tuberoinfundibular, mesocortical, and mesolimbic pathways. It has been shown that IG sugar injection can activate the nigrostriatal and mesolimbic dopaminergic pathways after several minutes.^22^^,^^23^^,^^24^^,^^25^^,^^26^^,^^27^ Early research focused on the projection targets of the nigrostriatal and mesolimbic dopaminergic pathways, namely the dorsal and ventral striatum, probably because the experimental techniques used, such as microdialysis and virus tracing, are more suited to detecting long-lasting dopaminergic reactions.^23^^,^^26^

On the other hand, recent research revealed that IG sugar injection activated the VTA, a component of the mesolimbic and mesocortical pathways, within seconds, as opposed to the several minutes it took to detect dopamine in the striatum. Based on the results of our local D1DR/D2DR inhibition experiments, it has become clear that the immediate activation of the frontal cortex following IG glucose injection is triggered by the direct action of dopamine on the frontal cortex. Considering those dopaminergic neurons project directly to the frontal cortex via the mesocortical pathway, the immediate activation of the frontal cortex that we observed suggests the involvement of the mesocortical pathway.^39^ This is significant because the dopamine-induced activation of the frontal cortex plays an important role in reward-based decision-making.

Previous reports suggest that the immediate activation of VTA is initiated by neuropod cells that transport glucose via SGLT1 and synapse with the left vagus nerve.^22^^,^^23^^,^^24^^,^^34^^,^^36^^,^^37^^,^^38^ In fact, our results demonstrated that IG glucose injection immediately activated the frontal cortex through SGLT1, the left vagus nerve, and a central dopaminergic pathway (Figures 2, 3, and S1). Moreover, since the frontal cortex did not activate following IG Ace K injection or intraperitoneal phlorizin pretreatment (Figures 2 and 3), the response would not occur due to sensory stimulation or osmolality. However, we did not observe frontal cortex activity when selectively inhibiting SGLT-1 (Figures 3A–3C). Thus, further experiments are needed to confirm that these responses originate from neuropod cells. Other mechanisms, such as the IPAN-vagus nerve pathway, could also be considered.^63^

### The physiological significance of the immediate dopamine-induced activation of both astrocytes and neurons in the frontal cortex after intragastric glucose injection

Using fiber photometry, we confirmed that both astrocytes and neurons in the frontal cortex activate immediately after IG glucose injection. Furthermore, our pharmacological experiments revealed that the dopamine-induced increase in Ca^2+^ levels in astrocytes and neurons occurred via D2DR and D1DR, respectively. It is known that astrocytes release ATP/adenosine as gliotransmitters upon dopamine activation through D2DR. These molecules, in turn, activate neuronal adenosine 1 receptors (A1 receptors), leading to a reduction in neuronal activity.^64^^,^^65^^,^^66^^,^^67^ Interestingly, the disruption of this astrocyte-dependent dopamine regulation in the frontal cortex is linked to the development of obsessive-compulsive spectrum disorders because of the dysregulation of the corticostriatal circuit.^40^ The corticostriatal circuit, which involves projection from frontal cortex neurons to the striatum, plays an important role in food-seeking behavior.^39^ Indeed, when we individually inhibited D1DR and D2DR exclusively in the frontal cortex, the duration of neuronal activation became significantly longer compared to other groups, accompanied by the elimination of astrocytic Ca^2+^ activation due to D2DR inhibition (Figure 5E Saline: sponta. Vs. IG (late), −0.05 ± 0.05 vs. 0.05 ± 0.14, t(4) = −3.10, *p* = 0.04; Raclopride: sponta. Vs. IG (late), 0.00 ± 0.026 vs. 0.33 ± 0.13, t(5) = 1.11, *p* = 0.32; SCH23390: sponta. Vs. IG (late), 0.10 ± 0.04 vs. −0.07 ± 0.06, t(3) = −0.59, *p* = 0.58).

Although our study did not directly demonstrate the release of ATP/adenosine following astrocyte activation via D2DR, or the subsequent suppression of neuronal activity, the observed increase in the intracellular Ca^2+^ level in astrocytes following dopamine activation via D2DR could be considered a key factor in modulating sugar intake in mice.

### Astrocytes in the frontal cortex respond to dopamine with different patterns

Our fiber photometric recordings following IG glucose injection revealed that while neurons exhibited a transient increase in fluorescence intensity, astrocytes exhibited a complex pattern in response to dopamine stimulation in the frontal cortex (Figure 4B). Specifically, we observed an initial transient increase in fluorescence intensity, followed by a reduction approximately 40 s after the injection. This decline was also noted after IG water injection and after dopamine antagonist treatment following IG glucose injection (Figures S3A–S3D). These observations led us to hypothesize that the reduction in astrocytic Ca^2+^ levels during the latter phase might be due to mechanisms unrelated to the dopamine response induced by IG glucose injection.

To further investigate these response patterns, we monitored the Ca^2+^ activity of individual astrocytes after IG glucose injection. We discovered diverse response patterns among frontal cortex astrocytes, indicating that the composite response observed with fiber photometry does not reflect synchronized activity across all responding cells (Figures 4F–4H and S6A–S6C). The patterns were primarily categorized into three types: [1] an increase in Ca^2+^ level, [2] a decrease in Ca^2+^ level, and [3] no response. The percentage of cells exhibiting decreased Ca^2+^ level was the most common pattern after IG glucose injection. The IG glucose injection group tended to show a higher percentage of cells exhibiting the decreased Ca^2+^ compared to the IG water injection group (Figure S6B, IG water vs. IG glucose, 0.21 ± 0.12 vs. 0.47 ± 0.24, *p* = 0.055). Conversely, the most common pattern observed in the group injected with intragastric (IG) water was no response. Additionally, the percentage of cells showing no response was higher in the IG water injection group compared to the IG glucose injection group (Figure S6C, IG water vs. IG glucose, 0.68 ± 0.14 vs. 0.30 ± 0.25, *p* = 0.013). At a cellular level, the onset of a Ca^2+^ decrease in astrocytes following IG injection was delayed by approximately 9 s compared with a Ca^2+^ increase, although this difference was not significant (Figures S6D–S6F).

We speculate that the increase in fluorescence intensity in some cells may arise from heterogeneity within the astrocyte population, a notion supported by previous observations of cultured astrocytes.^66^ As for the decrease in Ca^2+^ levels, it is likely not caused by metabolite-derived signals in the late phase after IG injection, but rather by other acute responses induced by the IG injection itself. The occurrence of an intracellular Ca^2+^ decrease in astrocytes is a compelling phenomenon, with many aspects that are not yet fully understood. Considering the diverse responses to the artificial activation of vagus nerve fibers, such as an immediate decrease in heart rate and alterations in cerebrospinal fluid, as well as contradictory findings such as the activation or inhibition of neurons in the frontal cortex, further exploration is necessary to identify the factors responsible for the acute decrease in astrocytic Ca^2+^ levels induced by the stimulation methods used in this study.^68^^,^^69^^,^^70^

### Chronic mild restraint stress decreases the immediate activation of the left vagus nerve after intragastric glucose injection

Chronic stress has been demonstrated to reduce sucrose preference in mice,^44^^,^^70^ suggesting alterations in reward processing mechanisms. Such stress is known to induce various changes within the brain, particularly affecting dopamine neurotransmission,^46^^,^^47^^,^^48^^,^^50^^,^^52^ and it also impacts the activity of the autonomic nervous system, including vagus nerve functions. The specific effects of stress on vagus nerve activities, including deviations in spike frequency bands and aberrant vagus nerve signals, are complex and warrant further investigation, as they may contribute to brain alterations.^49^^,^^51^^,^^71^

Our results showed that the peak response of the left vagus nerve after IG injection was not significantly different between the stressed mice receiving IG water and IG glucose. Furthermore, there was no significant difference in the IG water injection responses between stressed and healthy mice, although a modest but significant difference was noted in the IG glucose injection response between the two groups (Figures 6C and 6D, IG glucose injection + CMRS vs. IG glucose injection, 2.81 ± 1.56 vs. 1.34 ± 1.03, *p* = 0.06). On the basis of these observations, we hypothesized that CMRS-exposed mice might exhibit reduced activation of the frontal cortex in response to IG glucose injection. This hypothesis was confirmed by the observation of a significant reduction in frontal cortex activation in CMRS-exposed mice in response to IG glucose injection, with no significant difference in terms of the response to IG water injection (Figures 6F and 6G).

The role of dopamine-induced frontal cortex activation in reward-based decision-making is well established.^35^^,^^36^^,^^37^^,^^38^^,^^39^ Our results suggest that the decreased sucrose preference in CMRS-exposed mice could be partially attributed to the reduced activation of the left vagus nerve, possibly by neuroepithelial cells. However, our experiments did not verify whether the decrease in sucrose preference could be ameliorated by specifically activating neuroepithelial cells in CMRS-exposed mice. Previous research showed that specific signal activation in neuroepithelial cells during a test involving the choice of sucrose (which contains glucose) or sucralose (which does not contain glucose) led to a stronger preference for glucose-containing sucrose in comparison to sucralose.^30^ As such, further studies are needed to directly investigate the contribution of neuroepithelial cell–derived signals to the reduced sucrose preference observed in CMRS-exposed mice.

Our study demonstrated that IG (duodenal) glucose injection activated the left vagus nerve and frontal cortex within seconds; this was predicted to originate from neuropod cells that synapse with the vagus nerve almost instantaneously. Additionally, we observed that frontal cortex activation levels immediately following IG glucose injection were diminished after CMRS, which may be due to CMRS-induced abnormalities in vagus nerve activities.

The activation of the frontal cortex by dopamine plays an important role in reward-based decision-making and the regulation of striatal activity by modulating corticostriatal circuit connectivity.^35^^,^^36^^,^^37^^,^^38^^,^^39^^,^^40^ While our research concentrated on the ventral dopaminergic pathway, many studies have focused on the dorsal striatum following dorsal dopaminergic pathway activation and have shown that dopamine uptake in the dorsal striatum after gastric glucose absorption stimulates sugar intake in mice.^18^^,^^23^^,^^26^^,^^32^^,^^43^

We have not yet determined if frontal cortex activation directly enhances sucrose intake in mice, or if frontal cortex activation–induced changes in the activity of other brain regions, such as the striatum, indirectly facilitate sucrose intake. Nonetheless, our results suggest that the immediate activation of the frontal cortex following glucose absorption in the gut is crucial for the sucrose preference in mice. Furthermore, any abnormalities in this activation process may contribute to the reduced sucrose preference observed in mice subjected to chronic stress.

### Limitations of the study

In this study, there are several aspects that we were unable to investigate.

The first point is the involvement of the VTA in the activation of the frontal cortex. By recording Ca^2+^ signals with D1R/D2R blockers, we observed that both neurons and astrocytes were activated in response to dopamine. Considering previous reports indicating that intragastric (IG) sugar injection rapidly activates the VTA, it is speculated that the frontal cortex activation occurs via the mesocortical pathway.^22^ However, we have not yet confirmed whether the activation of the frontal cortex is directly mediated by the VTA.

The second point concerns the involvement of neuropod cells. Given the timescale and the role of SGLT1 in both vagus nerve and frontal cortex activation, it is plausible that these phenomena are mediated by neuropod cells.^23^^,^^32^^,^^33^^,^^34^ However, we have not directly investigated the involvement of neuropod cells in this process.

The final point is that we have not identified the factors contributing to the decrease in astrocytic Ca^2+^ levels following IG sugar injection. As research on the mechanisms underlying astrocytic Ca^2+^ level reduction is currently limited, we were unable to conduct experiments specifically addressing this issue.

## Resource availability

### Lead contact

Requests for further information and resources should be directed to and will be fulfilled by the lead contact, Hiromu Monai (monai.hiromu@ocha.ac.jp).

### Materials availability

This study did not generate new unique reagents.

### Data and code availability

• Data: All value used in the figure and the raw data of Figures 1, 2, 3, and 6G are deposited at https://data.mendeley.com/datasets/b8fp5fr44v/1. Accession numbers are listed in the key resources table.
• Code: Original code used to analyze transcranial Ca^2+^ imaging data and two-photon imaging data are deposited at https://data.mendeley.com/datasets/b8fp5fr44v/1. Accession numbers are listed in the key resources table. Detail steps of data analysis are shown in the method details section.
• Additional information: Any additional information in this article is available from the lead contact upon request.

## Acknowledgments

This work was supported by Ochanomizu University, the RIKEN CBS-EVIDENT Open Collaboration Center (BOCC), 10.13039/501100001691KAKENHI grants (18K14859, 20K15895), the JST FOREST Program (Grant Number JPMJFR204G), the 10.13039/501100012018Research Foundation for Opto-Science and Technology, the Kao Research Council for the Study of Healthcare Science, the 10.13039/100012040Japan Association for Chemical Innovation, and the TERUMO LIFE SCIENCE FOUNDATION. We extend our gratitude to Takashi Shichita of Tokyo Medical and Dental University for generously providing AAV9-CaMKIIa-jGCaMP7f, and to Atsushi Miyawaki from the RIKEN Center for Brain Science for his supervision of two-photon imaging. We also acknowledge that some of the figures were created using BioRender (https://www.biorender.com/). We would like to extend our gratitude to Dr. Toshiaki Teratani, Division of Gastroenterology and Hepatology, Department of Internal Medicine, Keio University School of Medicine, Tokyo, Japan, for his invaluable guidance and insights regarding the anatomical aspects of the vagus nerve.

## Author contributions

SY conducted all physiological experiments and performed data analysis. AN provided the resource, particularly for the fiber photometry recording and its analysis. KH and TT contributed to the conceptualization of the study and participated in the writing, review, and editing of the article. HM managed project administration, supervision, and funding acquisition.

## Declaration of interests

The authors declare no competing interests.

## Declaration of generative AI and AI-assisted technologies in the writing process

During the preparation of this work, the authors used ChatGPT to check grammar and polish the article. The authors reviewed, corrected, and edited the generated contents as needed.

## STAR★Methods

### Key resources table

**Table:** 

REAGENT or RESOURCE	SOURCE	IDENTIFIER
**Antibodies**		

Mouse monoclonal anti-CaMKⅡalpha	abcam	ab22609
Rabbit polyclonal anti-GFP	Thermo Fisher Scientific	Cat#A-6455; RRID: AB_217500
Goat polyclonal anti-GFP	ROCKLAND	Cat#600-101-215; RRID: AB_218182
Goat anti-Mouse IgG secondary antibody, Alexa Fluor™ 594	Thermo Fisher Scientific	Cat#A-11005; RRID: AB_2534073
Goat anti-Rabbit IgG secondary antibody, Alexa Fluor™ 488	Thermo Fisher Scientific	Cat#A-11008; RRID: AB_143165
Donkey anti-Goat IgG secondary antibody, Alexa Fluor™ 488	Thermo Fisher Scientific	Cat#A-11055; RRID: AB_2534102

**Bacterial and virus strains**		

AAV9-CaMKIIa-jGCaMP7f	Natsubori, A et al.^72^	N/A

**Chemicals, peptides, and recombinant proteins**		

Phlorizin	MedChemExpress	60-81-1
SCH23390	Sigma	125941-87-9
Raclopride	Sigma	98185-20-7
Glucose	Wako	50-99-7
Sucrose	Wako	57-50-1
Isoflurane	Viatris	871119
Saline	Otsuka Pharmaceutical Factory	35061311
Cyanoacrylate glue (AronArufa A)	TOAGOSEI	7990700Q1022
dental acrylic cement C&B	Sun Medical	221AABZX00115000
Depilatory cream	Kracie	N/A
Fluo4-AM	Thermo Fisher Scientific	F23917
Sulforhodamine 101	Sigma	60311-02-6
DMSO	Sigma	67-68-5
Pluronic F127	Sigma	9003-11-6
Agarose-LM	Nacalai tesque	9012-36-6

**Deposited data**		

Raw data and Value in all figures	This paper	Medeley: https://doi.org/10.17632/b8fp5fr44v.1
Source code	This paper	Medeley: https://doi.org/10.17632/n2v7bw8rg6.1

**Experimental models: Organisms/strains**		

Mouse; G7NG817	RIKEN Bio Resource Center	RBRC09650
Mouse; Mlc-YC (Mlc-tTA::tetO-yellow Cameleon-Nano50)	Kanemal et al.^73^	RBRC09550
Mouse; C57BL/6J	The Jackson Laboratory	Strain #:000664

**Software and algorithms**		

MATLAB	MathWorks	R2023a
ImageJ	Schneider et al.^74^	https://imagej.nih.gov/ij/
Labview	National Instruments	N/A
HCImage	Hamamatsu Photonics	ORCA-Spark
OriginPro 2024b	LightStone	https://www.originlab.com/index.aspx?go=Products/Origin/2024b&pid=5464

**Other**		

Cuffs electrodes	Unique Medical	N/A
Thin glass coverslip	Matsunami Glass	17-02-28
Drug-administration guide cannula	Eicom	CXG-4T
Drug-administration cannula	Eicom	CXMI-4T
Drug-administration guide cannula tube	Eicom	JF-10
Infusion pump	Melquest	FP-1100

### Experimental model and subject details

#### Animals

All experimental protocols were approved by the Institutional Animal Care and Use Committee of Ochanomizu University, Japan (approval No. 23006). All animal experiments were performed according to the guidelines for animal experimentation of Ochanomizu University, which in turn conform with the Fundamental Guidelines for Proper Conduct of Animal Experiments and Related Activities in Academic Research Institutions (Ministry of Education, Culture, Sports, Science and Technology, Japan). Efforts were taken to minimize the number of animals used. This study was carried out in compliance with the ARRIVE guidelines.

Adult male and female wild type, G7NG817 transgenic, and Mlc-YC (Mlc-tTA::tetO-yellow Cameleon-Nano50) double transgenic mice (older than 8 weeks) were used.^54^^,^^72^ The background strain of all mice was C57BL/6. Mice were housed under a 12-h/12-h light/dark cycle and raised in groups of up to five mice each. G7NG817 mice were obtained from the RIKEN Bio Resource Center (Resource IDs: RBRC09650).

### Method details

#### Catheter insertion into the proximal duodenum

Under 2.0% isoflurane anesthesia, the catheter insertion procedure was conducted as previously described.^75^ Mice were placed on a surgical table with a heat pad maintained at 37°C. A skin incision of approximately 1.5 cm was made 1 cm to the right of the abdominal median and 5 mm below the xiphoid process. Then, a 1.5-cm incision was created in the abdominal wall at the same location as the skin incision. A small perforation (approximately 1.5 mm in diameter) was made in the pyloric antrum, and the catheter tip was inserted. The catheter was secured and the abdominal wall was sutured using a 5/0 silk suture, allowing the catheter to protrude externally. The skin incision was then sutured in a similar manner to the abdominal closure. The catheter was flushed with saline and sealed with a needle cap to prevent bacterial infection. Mice were then returned to their cage for a minimum recovery period of 48 hours.

#### Preparation for *in vivo* transcranial Ca^2+^ imaging

Under 2.0% isoflurane anesthesia, preparation for *in vivo* transcranial Ca^2+^ imaging was performed a day prior to the imaging session as previously outlined.^75^ The mouse was positioned on a stereotaxic platform, and the scalp hair was removed using hair removal cream. After applying local anesthetic gel, the scalp was completely excised. The connective tissue of the periosteum was removed, and acrylic cement was promptly applied to the skull to prevent opacity caused by evaporation. The skull surface was allowed to dry for 5 minutes.

#### Optical fiber implantation

Stereotaxic surgery was performed under anesthesia with a ketamine–xylazine mixture (100 and 10 mg/kg injected intraperitoneally, respectively). For fiber photometric recordings, an optical fiber cannula (CFMC14L05, ⌀ 400 mm, 0.39 NA, Thorlabs) was implanted into layer 5 of the right secondary motor cortex (M2) in one direction (anteroposterior (AP), +1.8 mm; mediolateral from bregma (BL), 0.7 mm; dorsoventral from the skull surface (DV), 1.4 mm).

#### Surgical procedure for fiber photometry with local drug administration

Stereotaxic surgery was performed under anesthesia with a ketamine–xylazine mixture (100 and 10 mg/kg injected intraperitoneally, respectively). To record neural and astrocytic Ca^2+^ signals following local drug administration, a drug-administration guide cannula (CXG-4T, Eicom), along with an optical fiber (CFMC14L10, ⌀ 400 μm, 0.39 NA, Thorlabs), was inserted into the layer 5 of the right secondary motor cortex (M2) in one direction (anteroposterior (AP), +1.8 mm; mediolateral from bregma (BL), 0.7 mm; dorsoventral from the skull surface (DV), 1.4 mm).

#### Virus injection

For microinjection of adeno-associated virus (AAV) into the brain for fiber photometric recording, AAV9-CaMKIIa-jGCaMP7f (3.0 × 10^13^ vg per mL)^73^ was injected into layer 5 of the right secondary motor cortex (AP, +1.8 m; ML, 0.7 mm; DV, 1.4 mm). AAV microinjection was performed using a stainless-steel microinjection cannula (CXMI-4T, Eicom) attached to a 10-mL Hamilton syringe directed by a syringe pump (Legato130, KD Scientific) at a flow rate of 0.04 mL/minute for 10 minutes. AAV microinjection was carried out 3 weeks before surgery for fiber implantation.

#### Surgery procedures for two-photon imaging

For two-photon imaging, craniotomy with a diameter of 2 mm was performed above the frontal cortex (AP, +1.65 mm, ML, 1.35 mm). The dura mater was surgically removed. Fluo4-AM (Thermo Fisher Scientific, 1 μM, dissolved in a DMSO stock solution containing 10% Pluronic F127) was topically applied for 2 hours to facilitate Ca^2+^ activity detection, followed by Sulforhodamine 101 (100 μM in saline) for 1 minute to specifically label astrocytes. Sulforhodamine 101 was then rinsed with HEPES-buffered artificial cerebrospinal fluid (aCSF). After the dye loading, the craniotomy site was covered with agarose (2.0% w/v in aCSF) and gently sealed with a thin glass coverslip (2.7 mm × 2.7 mm, thickness: 0.3 mm, Matsunami Glass). The cranial window was then secured to the skull using dental cement.

#### Surgical procedure for a cervical vagus nerve electrophysiological recording

Cuff electrodes covered with a silicon tube (outer diameter: 0.7 mm; inner diameter: 0.3 mm, Unique Medical) and connected to gold pins or plastic connectors were attached to the left cervical vagus nerve. Under 2.0% isoflurane anesthesia, mice in which a catheter was previously attached to the gut were secured on a surgical table equipped with a heating pad maintained at 37°C. The neck hair was removed using hair removal cream, and then local anesthetic gel was applied. The ventral cervical area was incised using scissors, and the salivary glands were cautiously removed using tweezers. The left vagus nerve was carefully dissected from the carotid sheath, and the cuff portions of the electrodes were attached to the nerve.

#### Surgical procedure for a vagus nerve ablation

Under 2.0% isoflurane anesthesia, mice were secured on a surgical table equipped with a heating pad maintained at 37°C. Local anesthetic gel was applied to the upper left abdominal area. The upper left abdominal area was incised using scissors, and then, the stomach and the upper part of the esophagus were exposed. The left vagus nerve projecting to the somach along the abdominal esophagus was identified at the junction between the esophagus and the stomach. The nerve was ablated at this location using fine forceps. The skin incision was then sutured in a similar manner to the abdominal closure. The catheter was flushed with saline and sealed with a needle cap to prevent bacterial infection. Mice were then returned to their cage for a minimum recovery period of 48 hours.

#### Behavioral test

##### Preference test: sucrose vs. water and glucose vs. water

Before CMRS, pairs of mice were systematically paired while ensuring gender segregation. These pairs were then introduced into cages containing two bottles, designed to mitigate spillage through vibrations. To familiarize the mice with the presence of the bottles, both were initially filled solely with water for several days. One day prior to the start of the preference test, one bottle was filled with a sugar solution (1% sucrose solution for the sucrose preference test, or 3% glucose solution for the glucose preference test), while the other remained filled with water. Mice were granted unrestricted access to both bottles. On the day of the test, the weights of both bottles were measured, and their positions within the cage were alternated from the previous day. One day into the preference test, their positions were swapped to counteract any side preference bias in the mice. Two days into the preference test, the weights of both bottles were recorded. One day after the CMRS procedure, each bottle, containing either a sugar solution (sucrose or glucose) or water, was positioned in the cages where the stress procedure had been conducted. Mice were given free access to both bottles; following the protocol established in the pre-stress test, the positions of the bottles were switched 1 day after the test began. Two days after the start of the test, the bottles were collected and their weights were measured. The preference for the 1% sucrose or 3% glucose solution was calculated as follows.

$$\frac{\text{sucrose}\;\text{or}\;\text{glucose}\;\text{consumption}\;\left[\text{mL}\right]}{\text{total}\;\text{fluid}\;\text{consumption}\;\left[\text{mL}\right]}\times 100\text{.}$$

##### Preference test: Measuring the sucrose preference of each group of mice following saline, raclopride, or SCH23390 intraperitoneal pretreatment

Pairs of mice were systematically paired while ensuring gender segregation. These pairs were then introduced into cages containing two bottles, designed to mitigate spillage through vibrations. To familiarize the mice with the presence of the bottles, both were initially filled solely with water for several days. Two days prior to the start of the preference test, one bottle was filled with a 1% sucrose solution, while the other remained filled with water. Mice were granted unrestricted access to both bottles. At 8:00 PM on the day before the first test day, only the bottle filled with a 1% sucrose solution was removed. At 2:45 PM on the first test day, the weights of bottles filled with water or 1% sucrose solution were measured. At 3:00 PM, two bottles were placed in each cage. At 8:00 PM, both bottles were collected, and their weights were recorded. Subsequently, only the bottle containing water was returned to each cage. At 2:40 PM on the second test day, the weights of bottles filled with water, or 1% sucrose solution were measured, and at 2:45 PM, mice were intraperitoneally administrated saline, raclopride, or SCH23390. At 3:00 PM, two bottles were placed in each cage with their positions alternated from the previous day. At 8:00 PM, both bottles were collected, and their weights were recorded. The preference for the 1% sucrose was calculated as follows.

$$\frac{\text{sucrose}\;\text{or}\;\text{glucose}\;\text{consumption}\;\left[\text{mL}\right]}{\text{total}\;\text{fluid}\;\text{consumption}\;\left[\text{mL}\right]}\times 100\text{.}$$

##### CMRS procedure

Mice were paired with same-sex counterparts and placed together in a small cage for 8 hours a day for 10 consecutive days. The cage was a plastic circular container (diameter: 7 cm, depth: 8 cm) with several small holes to allow for ventilation. Throughout the CMRS procedure, each mouse had restricted access to water, but their food intake was unrestricted, as pellets were placed in the container.

#### Intraperitoneal drug application

The SGLT1 inhibitor phlorizin was dissolved in saline (100 mM, MedChemExpress) and intraperitoneally injected at a dose of 100 μg/kg 20 minutes prior to initiating transcranial cortex-wide Ca^2+^ imaging or vagus nerve electrophysiological recordings. The D1DR antagonist SCH 23390 (0.25 mg/kg, Sigma) was dissolved in saline and administered intraperitoneally 30 minutes before commencing transcranial cortex-wide Ca^2+^ imaging and fiber photometric recordings. The D2DR antagonist raclopride (400 μg/kg, Sigma) was dissolved in saline or administered intraperitoneally 20 minutes before starting transcranial cortex-wide Ca^2+^ imaging or fiber photometric recordings.

#### Local drug application

To administer the drug locally, raclopride (5 nmol, Sigma) or SCH 23390 (8 nmol, Sigma) was first dissolved in saline. Each solution was then drawn into the drug-administration cannula, which was connected to a 25-μL Hamilton syringe via an FEP tube (JF-10, Eicom). The 25-μL Hamilton syringe was attached to an infusion pump (FP-1100, Melquest) to control the infusion rate. Under 2.0% isoflurane anesthesia, the drug-administration cannula was inserted into the site where the guide cannula had been placed. Subsequently, 4 μl of each solution was locally administered to the M2 region of each group of mice at a rate of 0.2 μl/min through the drug-administration cannula. Fiber photometric recordings were conducted 30 minutes after the start of the local injection.

#### *In vivo* transcranial cortex-wide Ca^2+^ imaging

G7NG817 mice under 1.0% isoflurane anesthesia were placed on a stereotaxic platform using auxiliary ear bars under a fluorescence stereo microscope (MVX10, Evident). A U-MGFPHQ filter set (excitation 460–480 nm, emission 495–540 nm, Evident) was used with a U-HGLGPS light source (Evident). Images with a size of 512 × 512 pixels (16-bit resolution) were acquired at 10 Hz using an ORCA-spark CMOS camera (Hamamatsu Photonics) and HC Image software (Hamamatsu Photonics). Under 1.0% isoflurane anesthesia, stable Up/Down states were observed over an extended period.

#### *In vivo* two-photon imaging

Two-photon imaging was performed on 1.0% isoflurane-anesthetized wild-type mice using a multi-photon laser scanning microscope (FVMPE-RS, Evident) equipped with an InSight laser system (Spectra-Physics) and an Olympus objective (FV30-AC25W, numerical aperture: 1.05, working distance: 2 mm, immersion medium: water). Images obtained during IG injection sessions were acquired at 10 Hz with a size of 512×512 pixels (16-bit resolution) using a resonant scanner, whereas single images before each IG injection session were acquired using a galvano scanner at the same resolution to identify cells containing merged green and red signals. The excitation wavelength was adjusted from 690 to 1,100 nm. Laser emission wavelengths of 495–540 nm were used for Fluo4-AM, while wavelengths of 575–645 nm were used for SR101 excitation, in both cases employing an FV30-FGR filter (Evident). Images were captured using Evident software.

#### Fiber photometry

To detect Mlc-YC and CaMKII-GCaMP7f fluorescence signals, a fiber photometric system custom-made by Olympus Engineering was used.^76^ For recording of Mlc-YC signals, input light (center wavelength of 435 nm; silver-LED-435, Prizmatix) was reflected off a dichroic mirror (DM455CFP, Olympus), linked to an optical fiber (M41L01, ⌀ 600 mm, 0.48 NA, Thorlabs), then linked to an optical fiber (M79L01, ⌀ 400 mm, 0.39 NA, Thorlabs) through a pinhole (⌀ 600 mm). It was then delivered to an optical fiber cannula (CFMC14L05, Thorlabs) implanted into the mouse brain. The LED power was < 200 μW at the fiber tip. Emitted yellow and cyan fluorescent light from the YC probe was collected via an optical fiber cannula, divided by a dichroic mirror (DM515YFP, Olympus) into cyan (483/32 nm band-pass filter, Semrock) and yellow (542/27 nm band-pass filter, Semrock) fluorescence, and detected using two distinct photomultiplier tubes (H7422-40, Hamamatsu Photonics). For recording of CaMKII-GCaMP7f signals, input light (center wavelength of 475 nm; silver-LED-475, Prizmatix) and dichroic mirrors (DM490GFP, Olympus and FF552-Di02–25x36, Semrock) were used to detect green fluorescence with a band-pass filter (BA495-540GFP, Olympus). For recording of GCaMP7f isosbestic control signals, input light (center wavelength of 405 nm; M405F1, Thorlabs) and dichroic mirrors (DMLP425R, Thorlabs; DM490GFP, Olympus; and FF552-Di02–25x36, Semrock) were used to detect green fluorescence with a band-pass filter (BA495-540GFP, Olympus). Fluorescent signals were digitized using a data-acquisition module (NI USB-6008, National Instruments) and recorded using a custom-made LabVIEW program (National Instruments). CaMKII-GCaMP7f and Mlc-YC signals were collected at 1 Hz and 1 kHz, respectively.

#### Vagus nerves electrophysiological recording

Vagus nerves electrophysiological recording was performed in mice under 1.0% isoflurane anesthesia using a heating pad maintained at 37°C, with a cuff electrode and a signal amplifier (HAS-4 Head Amplifier System, Bio Research Center). Data were amplified via a head stage (×1,000, filtered 10–7,000 Hz) and subsequently digitized using LabVIEW (National Instruments).

#### Image processing

##### Transcranial Ca^2+^ imaging analysis

For transcranial images, images were binned to 64 × 64 pixels, and hand-drawn regions of interest (ROIs) were determined using ImageJ software (NIH). ROIs were aligned with the mouse brain atlas and designated as the frontal area (Frontal), the somatosensory area including the barrel area (Somato), the occipital area (Visual), and the retrosplenial region (RSC). ROI coordinates were extracted using the MATLAB function “ReadImageJROI.” The average fluorescence intensity change rate (ΔF/F) within each ROI was computed using MATLAB, where ΔF/F was defined as follows.

$$\frac{\Delta F}{F}=\frac{\left(F_{t}-F_{0}\right)}{F_{0}}$$

where $F_{t}$ represents the fluorescence intensity value at a specific time and $F_{0}$ represents the average intensity value during the spontaneous state, defined as the 50 seconds preceding the injection start. The average fluorescence intensity change rate of each ROI underwent low-pass filtering to eliminate frequencies over 1 Hz, and Ca^2+^ oscillation peaks exceeding the baseline intensity value of each ROI were identified by MATLAB. Peak ratios for the Frontal, Somato, Visual, and RSC areas were determined by dividing each peak value by the peak value of the reference ROI (i.e., the auditory area). The spontaneous peak ratio was the average over 50 seconds before the start of the injection, and target waveforms were identified between 3 and 8 seconds after injection initiation. Activation levels were defined as the ratio of the peak ratio during injection to the spontaneous peak ratio.

For the analysis of peak widths, changes in Ca^2+^ dynamics in the Frontal cortex that exceeded 2ΔF/F were defined as Ca^2+^ oscillations. Then, peak widths of each Ca^2+^ oscillations were calculated by MATLAB. The average peak widths during spontaneous state (−50 to 0 s from IG injection) and later phase (10 to 60 s from IG injection) were computed for each mouse (Figures 2, 3, and S3).

##### Two-photon Ca^2+^ imaging analysis

For two-photon imaging analysis, ROIs were determined by manually outlining the merged areas in both SR101- and Fluo4-AM–positive cells. ROI coordinates were extracted using the MATLAB function “ReadImageJROI.” The average fluorescence intensity change rate (ΔF/F) within each ROI was computed using MATLAB, with ΔF/F defined as above. The average and standard deviation (SD) during the spontaneous state were computed. ROIs exhibiting fluorescence intensity exceeding the spontaneous average + 0.5 SD within 15–38 seconds after injection were labeled as “Increase” (this period was defined as the average duration of the increase in the populational astrocyte Ca^2+^ response in the frontal cortex). ROIs with a fluorescence intensity below the spontaneous average − 0.5 SD during the same timeframe were labeled as “Decrease.” ROIs not meeting either criterion were labeled as “Nonresponse.” The proportion of Increase, Decrease, and Nonresponse ROIs in each individual was determined on the basis of the count of each ROI type relative to the total number of ROIs per individual (Figures 4G and S6B). The average waveforms of Increase and Decrease ROIs in individuals in the IG glucose injection group were calculated; these values were then used to compute the peak onset, HWHM, and peak time for each individual (Figures S6D–S6F).

##### Fiber photometric imaging analysis

For neuronal and astrocytic Ca^2+^ upregulation analysis after IG injection, each individual’s ΔF/F was calculated. ΔF/F was defined as follows:

$$\frac{\Delta F}{F}=\frac{\left(F_{t}-F_{0}\right)}{F_{0}}\text{,}$$

where $F_{t}$ represents the fluorescence intensity value at a specific time and F_0_ represents the average intensity value in the spontaneous state, defined as the 30 seconds prior to the start of the injection. In the data from individuals subjected to IG glucose injection, the average and SD during the spontaneous state were calculated. The upregulation time post-injection, where the fluorescence intensity exceeded the average + 3 SD, was determined for each individual. The analysis window for post-injection fluorescence intensity was defined as the time between the minimum and maximum values of the upregulation time for the entire data of mice. The average waveform of each individual in the IG glucose injection group was calculated. Using the waveforms, the peak onset and HWHM were computed. In the neuron dataset, the difference between the median fluorescence intensity within the analysis window and the median fluorescence intensity during the spontaneous state was defined as the upregulation change in the neuronal Ca^2+^ level (Figures 4C and 5C). In the astrocyte dataset, the difference between the top 30% of the average fluorescence intensity values within the analysis window and the average fluorescence intensity during the spontaneous state was defined as the upregulation change in the astrocytic Ca^2+^ level (Figures 4F and 5E).

For the analysis of Ca^2+^ downregulation in astrocytes after IG injection, the downregulation duration after injection, where the fluorescence intensity was below the average − 3 SD, was determined for each individual. The analysis window for post-injection fluorescence intensity was defined as the time between the minimum and maximum values of the downregulation time for the entire data of mice. The difference between the average fluorescence intensity within the analysis window and the bottom 30% of the average fluorescence intensity during the spontaneous state was defined as the downregulation change in the astrocytic Ca^2+^ level (Figures S3B and S3D).

For the analysis of long-lasting neuronal Ca^2+^ upregulation, the average fluorescence intensity during the spontaneous state (−50 to 0 s from injection) and the later phase (30 to 80 s from injection) was calculated. These values were compared within each mouse.

##### Vagus nerve electrophysiological recording imaging analysis

For the analysis of vagus nerve electrophysiological recording, recording data were subjected to high-pass filtering, limiting the frequencies to those above 50 Hz. The filtered data’s average, maximum, and standard deviation in the spontaneous state (the 30 seconds prior to the start of the injection) were calculated, then the entire recording data were normalized with reference to the average and maximum values during the spontaneous state. The median and SD of the normalized data during the spontaneous state were calculated. Peaks exceeding the median + 2 SD were identified within the entire set of normalized data for each individual. Peaks surpassing the median + 5 SD were removed as noise. The number of peaks was calculated. The number of peaks per second was determined, and a comparison was made between the average number of peaks during the spontaneous state and that of peaks during the 2–4 seconds after the injection.

### Quantification and statistical analysis

Details on quantification and statistical tests are available in Method details and figure legends where applicable. The “n” indicated in the figure legend refers to the number of individuals (sample size) used in the experiment or study. The center box in each box plot figure represents the average value of each data. Upper and lower error bar in each box plot represent the maximum and minimum value of each data. All statical analyses were performed using OriginPro 2024b (LightStone, Japan). Statistical significances are shown as n.s., ^∗^, ^∗∗^, and ^∗∗∗^ indicate *P* < 0.05, *P* < 0.01, and *P* < 0.001, respectively. The file that includes all statistical value is available from Supplemental information.

## Supplemental information

Document S1. Figures S1–S6

Table S1. All statistics data
