# Supplementary material for: Immediate glucose signaling transmitted via the vagus nerve in gut–brain neural communication
Source: iScience. 2025 May 5;28(5):112439. doi: 10.1016/j.isci.2025.112439 (PMC12146620; doi:10.1016/j.isci.2025.112439)
Supplement: Document S1. Figures S1–S6 [file mmc1.pdf]

iScience, Volume *28*

Supplemental information

Immediate glucose signaling transmitted via the vagus nerve in gut–brain neural communication

Serika Yamada, Akiyo Natsubori, Kazuki Harada, Takashi Tsuboi, and Hiromu Monai

Figure S1

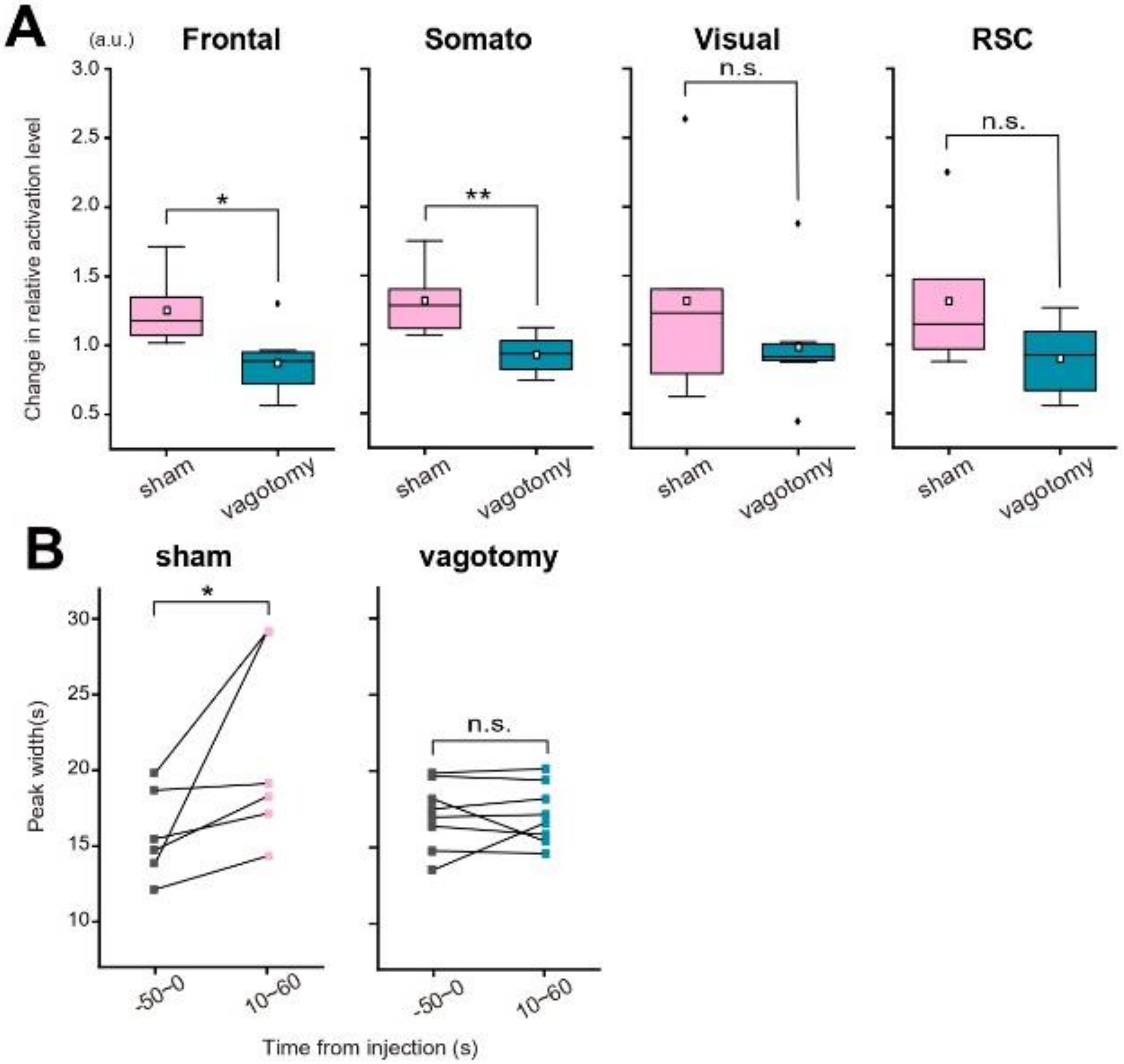

A
(a.u.)
Frontal
Somato
Visual
RSC
Change in relative activation level
3.0
2.5
2.0
1.5
1.0
0.5
*
**
n.s.
n.s.
sham
vagotomy
B
sham
vagotomy
Peak width(s)
30
25
20
15
10
*
n.s.
-50~0
10~60
Time from injection (s)

Figure S1 Vagus nerves transmit immediate glucose signal from gut to brain

(A) Comparison of activation levels in each cortical region of sham mice (left) and vagotomized mice (right) after IG glucose injection. The peak fluorescence intensity values in each brain region were compared against those of a reference region (auditory cortex) over a designated time window, including the 50 seconds before and after injection. Post-injection values were normalized by dividing them by the pre-injection average and compared between different treatment groups. $*p < 0.05$, $**p < 0.01$ (sham: n = 6 mice; vagotomy: n = 8 mice; two-sample t-test)

(B) Within-subject comparison of the widths of individual Ca^{2+} waves before (-50 to 0 s from injection) and in the later phase after IG glucose injection (10 to 60 s from injection) for each group (left: sham, right: vagotomy). $*p < 0.05$ (sham: n = 6 mice; vagotomy: n = 8 mice)

In each box plot, the central box shows the average (mean) value, while the horizontal line within the box represents the median. Box plot area represents the interquartile range (IQR). The upper and lower error bar in each box plot represent the maximum and minimum value of each data excluding outliers. Outliers were defined as data points that fall outside the range of 1.5 times the IQR and were represented as black points.

Figure S2

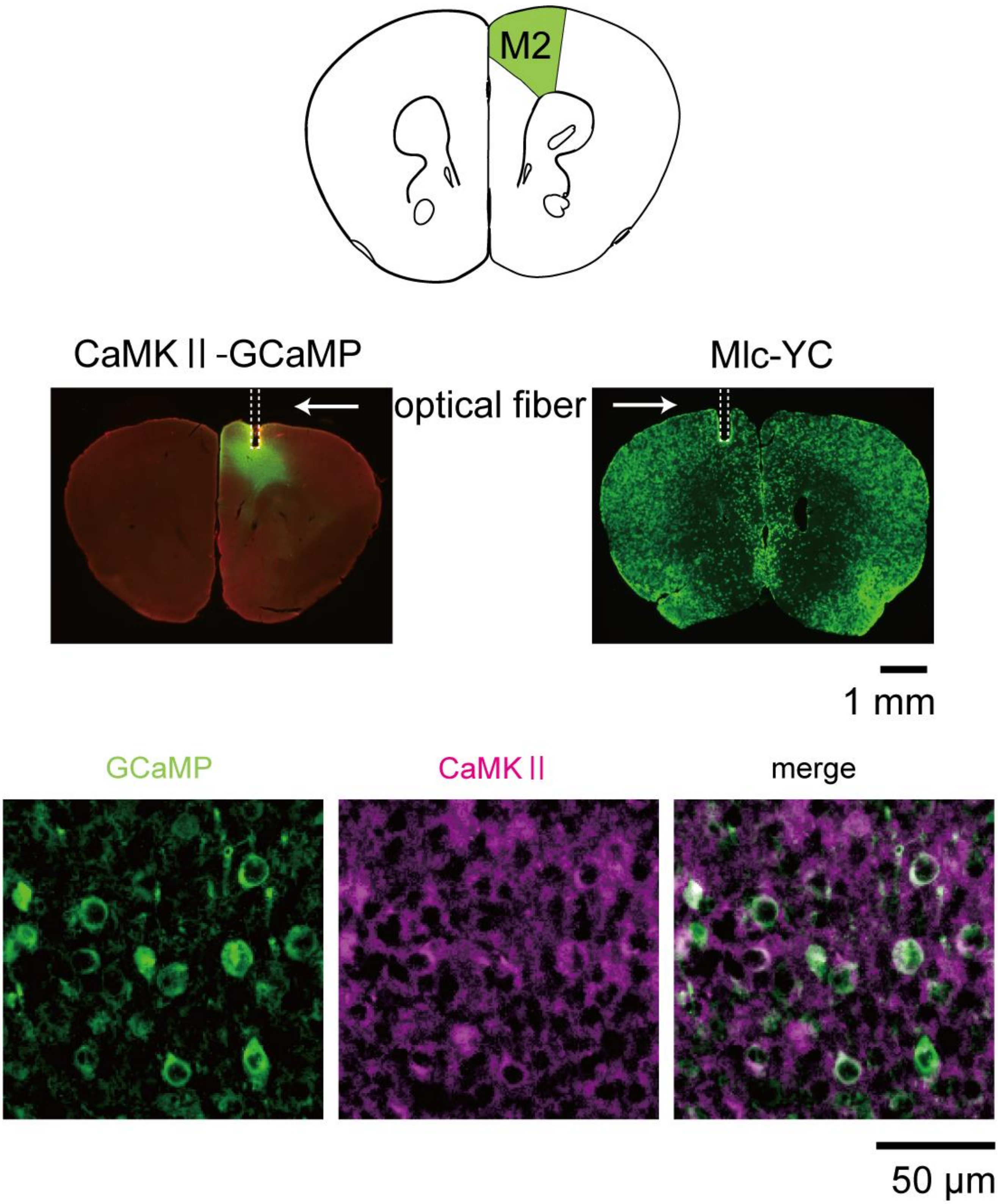
M2
CaMKII -GCaMP
Mlc-YC
optical fiber
1 mm
GCaMP
CaMKII
merge
50 µm

Figure S2 Histological Illustrations of CaMKII-GCaMP7f and Mlc-YC-nano50 probes

(top) Histological reconstructions depicting the placement of optical fiber tips in the secondary frontal cortex (M2) using CaMKII-GCaMP7f (left) and Mlc-YC (right) probes, with green indicating GFP staining. Scale bar: 1 mm. For the CaMKII-GCaMP7f probe, GFP immunostaining reveals CaMKII expression within pyramidal neurons. In Mlc-YC mice, GFP immunostaining indicates the presence of Yellow Cameleon Nano-50 driven by astrocyte-specific tetracycline transactivator expression.

(bottom) GFP fluorescence microscopy in layer 5 of M2, showing GCaMP7f (left), CaMKII (middle), and their composite image (right). Green denotes GCaMP7f fluorescence, while pink highlights CaMKII labeling. Scale bar: 50 μm.

Figure S3

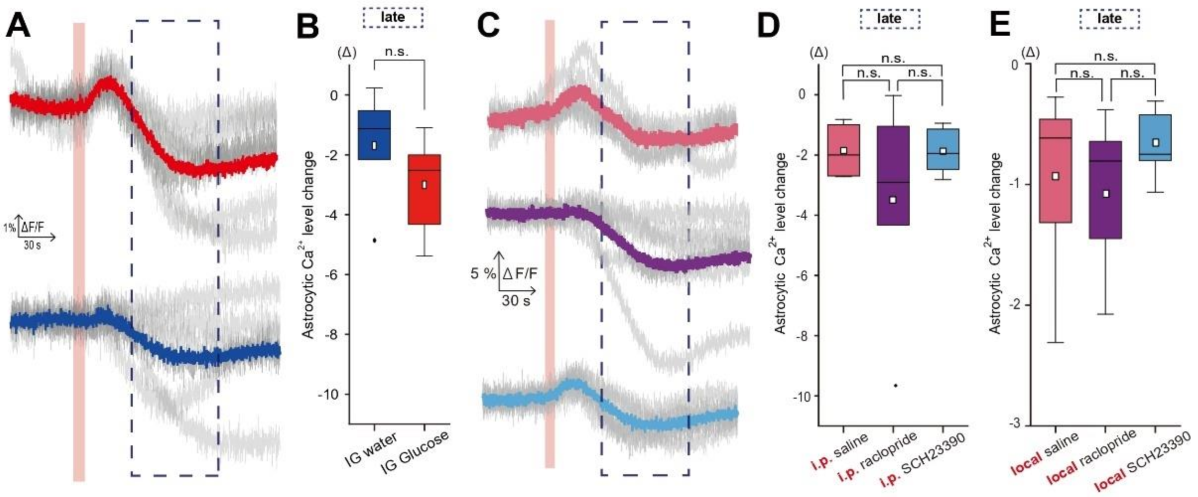

A
1% ΔF/F
30 s
B
late
(Δ)
n.s.
Astrocytic Ca2+ level change
0
-2
-4
-6
-8
-10
IG water
IG Glucose
C
5 % ΔF/F
30 s
D
late
(Δ)
n.s.
n.s.
n.s.
Astrocytic Ca2+ level change
0
-2
-4
-6
-8
-10
i.p. saline
i.p. raclopride
i.p. SCH23390
E
late
(Δ)
n.s.
n.s.
n.s.
Astrocytic Ca2+ level change
0
-1
-2
-3
local saline
local raclopride
local SCH23390

Figure S3 Late-phase astrocytic signal dynamics in M2 under IG glucose and water injections

(A) Traces of averaged Mlc-YC (Astrocytes) signal responses to IG glucose (red bold line) or IG water (blue bold line) injection, with the light gray line depicting traces from individual mouse. (glucose, n = 7 mice; water, n = 5 mice)

(B) Comparative analysis of late-phase changes in Mlc-YC (Astrocytes) signals in response to IG water (blue) or IG glucose (red) injection. The analysis window (late phase) corresponds to the area within the dotted line in (A). (water, n = 5 mice; glucose; n = 5 mice)

(C) Traces of averaged Mlc-YC (Astrocytes) signal responses to IG glucose injection following i.p. saline (pink bold line), raclopride (purple bold line), or SCH23390 (light blue bold line) injection, with the light gray line representing traces from individual mouse. (saline, n = 5 mice; raclopride, n = 8 mice; SCH23390, n = 7 mice)

(D) Comparative analysis of late-phase changes in Mlc-YC (Astrocytes) signals in response to IG glucose injection following i.p. saline (pink), raclopride (purple), or SCH23390 (light blue) injection. The analysis window (late phase) corresponds to the area within the dotted line in (C). (saline, n = 5 mice; raclopride, n = 8 mice; SCH23390, n = 7 mice)

In each box plot, the central box shows the average (mean) value, while the horizontal line within the box represents the median. Box plot area represents the interquartile range (IQR). The upper and lower error bar in each box plot represent the maximum and minimum value of each data excluding outliers. Outliers were defined as data points that fall outside the range of 1.5 times the IQR and were represented as black points.

Figure S4

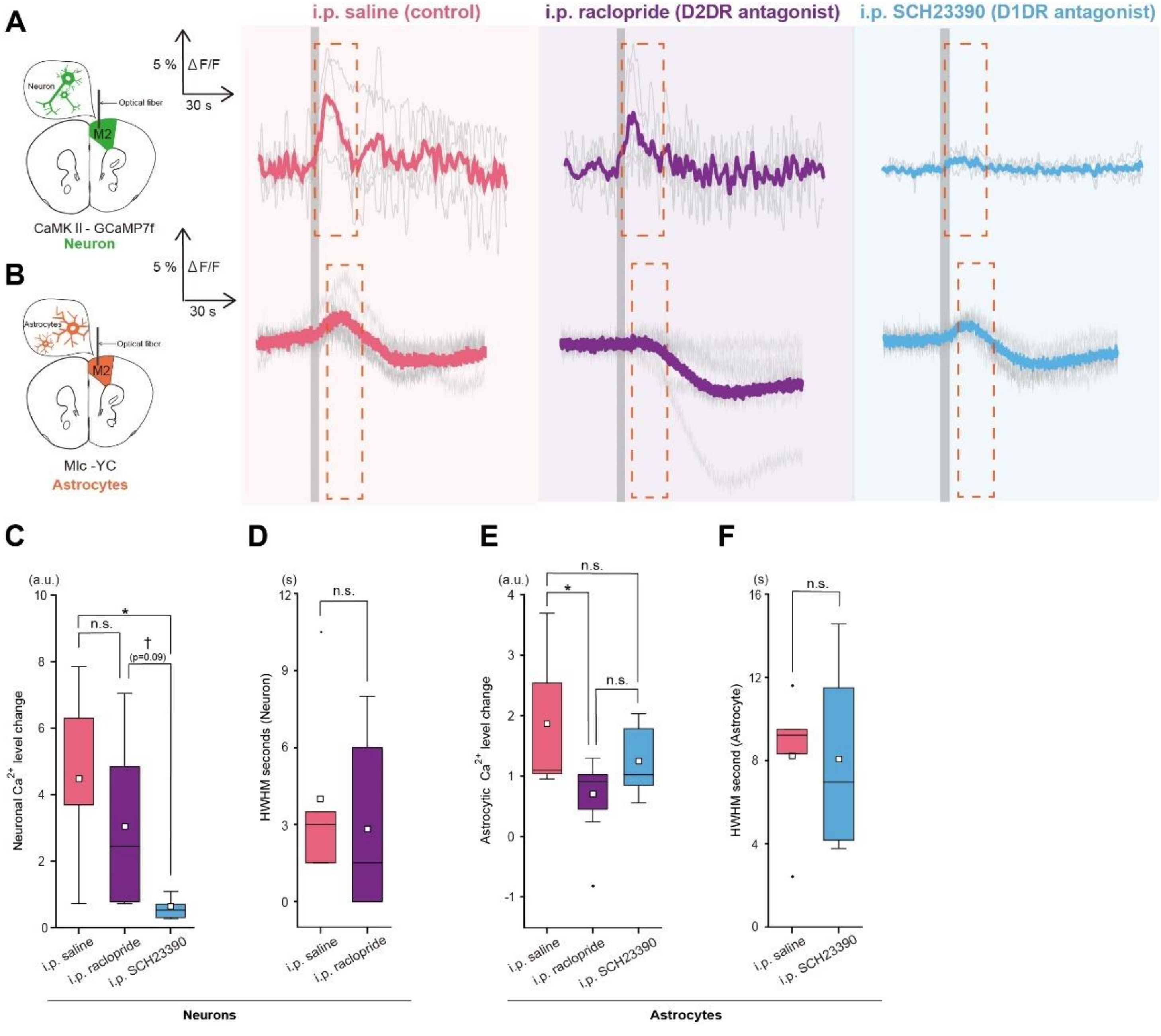
A
B
C
D
E
F
i.p. saline (control)
i.p. raclopride (D2DR antagonist)
i.p. SCH23390 (D1DR antagonist)
5 % ΔF/F
30 s
Neuron
Optical fiber
M2
CaMK II - GCaMP7f
Neuron
Astrocytes
Mlc -YC
Astrocytes
(a.u.)
Neuronal Ca2+ level change
n.s.
*
†
(p=0.09)
i.p. saline
i.p. raclopride
i.p. SCH23390
(s)
HWHM seconds (Neuron)
Astrocytic Ca2+ level change
HWHM second (Astrocyte)
Neurons
Astrocytes

Figure S4 Differential responses of neuronal and astrocytic signals to IG glucose injection modulated by dopamine.

(A) Fiber photometric traces of averaged CaMKII-GCaMP7f (Neurons) signal responses to IG glucose injection with saline (left), raclopride (middle), or SCH23390 (right) intraperitoneal pretreatment. The gray shaded area denotes the IG glucose injection period. (saline: n = 5 mice; raclopride: n = 6 mice; SCH23390: n = 5 mice)

(B) Fiber photometric traces of averaged Mlc-YC (Astrocytes) signal responses to IG glucose injection with saline (left), raclopride (middle), or SCH23390 (right) intraperitoneal pretreatment. The gray shaded area indicates the IG glucose injection period. (saline: n = 5 mice; raclopride: n = 8 mice; SCH23390: n = 7 mice)

(C) Comparison of Ca^{2+} activation levels after IG glucose injection of CaMKII-GCaMP7f (Neurons) in mice intraperitoneally pretreated with saline (left), raclopride (middle), or SCH23390 (right). †$p < 0.1$, *$p < 0.05$ (saline: n = 5 mice; raclopride: n = 6 mice; SCH23390: n = 5 mice; one-way ANOVA followed by Tukey–Kramer method)

(D) Comparison of the HWHM of CaMKII-GCaMP7f (Neurons) signals after IG glucose injection with saline (left) or raclopride (right) intraperitoneal pretreatment. (saline: n = 5 mice; raclopride: n = 6 mice; two-sample t-test)

In each box plot, the central box shows the average (mean) value, while the horizontal line within the box represents the median. Box plot area represents the interquartile range (IQR). The upper and lower error bar in each box plot represent the maximum and minimum value of each data excluding outliers. Outliers were defined as data points that fall outside the range of 1.5 times the IQR and were represented as black points.

Figure S5

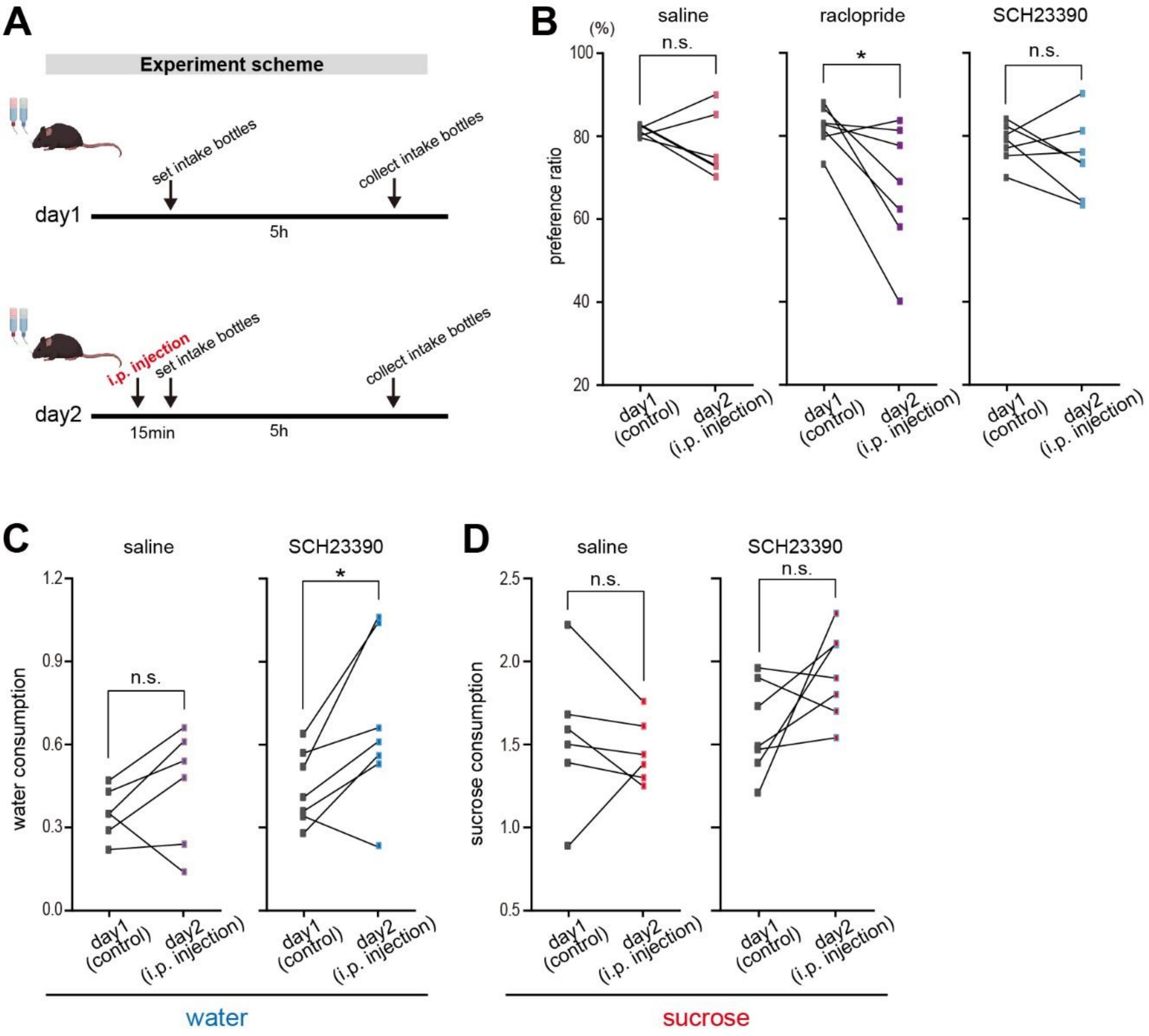
A
Experiment scheme
set intake bottles
collect intake bottles
day1
5h
i.p. injection
set intake bottles
collect intake bottles
day2
15min
5h
B
saline
raclopride
SCH23390
(%)
n.s.
*
n.s.
preference ratio
100
80
60
40
20
day1 (control)
day2 (i.p. injection)
C
saline
SCH23390
n.s.
*
water consumption
1.2
0.9
0.6
0.3
0.0
day1 (control)
day2 (i.p. injection)
water
D
saline
SCH23390
n.s.
n.s.
sucrose consumption
2.5
2.0
1.5
1.0
0.5
day1 (control)
day2 (i.p. injection)
sucrose

Figure S5 Impact of the inhibiting each dopamine receptor to sucrose preference

(A) Overview of the assessment to investigate the effects of dopamine antagonists (raclopride or SCH23390) on sucrose preference.

(B) Changes in the preference ratio for 1% sucrose solution between Day1 (control) and Day2 (intraperitoneal pretreatment) for each group. $*p < 0.05$ (saline: n = 6 group; raclopride: n = 7 group; SCH23390: n = 7 group; two-sample repeated t-test)

(C) Changes in the water consumption between Day1 (control) and Day2 (intraperitoneal saline or SCH23390 pretreatment) within each group. $*p < 0.05$ (saline: n = 6 group; raclopride: n = 7 group; two-sample repeated t-test)

(D) Changes in the 1% sucrose solution consumption between Day1 (control) and Day2 (intraperitoneal saline or SCH23390 pretreatment) within each group. (saline: n = 6 group; raclopride: n = 7 group; two-sample repeated t-test)

Figure S6

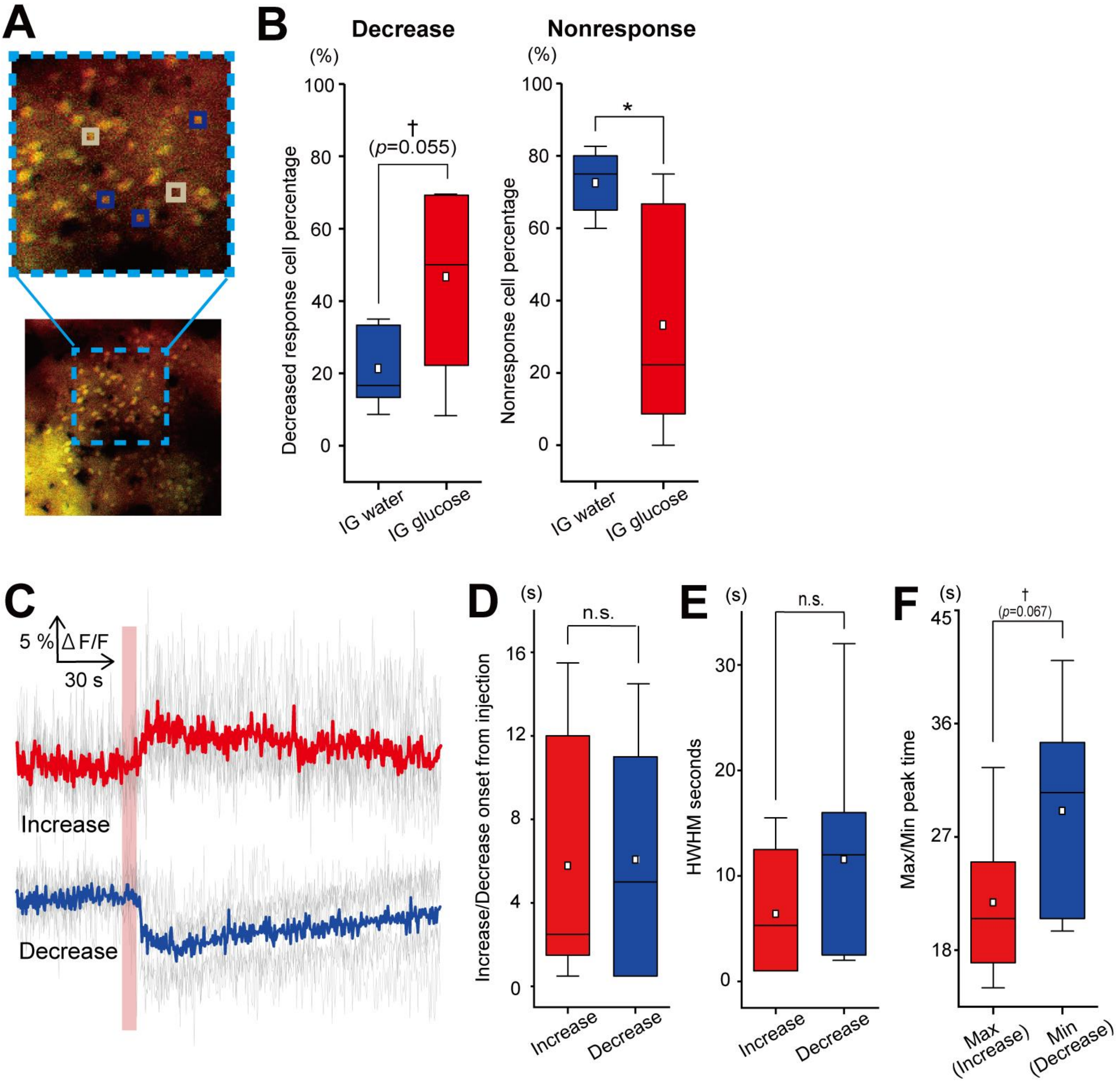

A
B
Decrease
Nonresponse
(%)
100
80
60
40
20
0
Decreased response cell percentage
† (p=0.055)
IG water
IG glucose
Nonresponse cell percentage
*
C
5 % ΔF/F
30 s
Increase
Decrease
D
(s)
n.s.
16
12
8
4
0
Increase/Decrease onset from injection
Increase
Decrease
E
HWHM seconds
30
20
10
F
† (p=0.067)
45
36
27
18
Max/Min peak time
Max (Increase)
Min (Decrease)

Figure S6 Late-phase, single-cell-level astrocytic signal dynamics in M2 after IG glucose injection

(A) Representative two-photon microscopy image of layer 2 in the frontal cortex, with cells labeled with Fluo4-AM (green, Ca^{2+} indicator) or SR101 (red, astrocytes). Colored squares mark ROIs; cells enclosed by blue squares exhibited a decreased response, while those surrounded by beige squares showed no response.

(B) Comparative analysis of the proportion of all evaluated cells in each mouse displaying a decreased response or no response. $^{**}p < 0.01$, $^{\dagger}p < 0.1$ (IG glucose, n = 7 mice; IG water, n = 5 mice; two-sample t-test)

(C) $\Delta F/F$ traces for the cell populations that exhibited an increase or decrease in fluorescence intensity after IG glucose injection (gray line), alongside the corresponding aggregate data (bold red and blue lines, respectively). (Increase, 25 cells from n = 7 mice; Decrease, 69 cells from n = 7 mice)

(D) Comparative analysis of the onset time of increased or decreased fluorescence intensity after IG glucose injection. (n = 7 mice, two-sample t-test)

(E) Comparative analysis of the HWHM for the average data of each mouse that exhibited an increase or decrease in fluorescence intensity. (n = 7 mice, two-sample t-test)

(F) Comparison of the maximum and minimum peak times of the fluorescence intensity in cells exhibiting increased or decreased fluorescence intensity, respectively, under IG glucose injection. $^{\dagger}p < 0.1$ (n = 7 mice, two-sample t-test)

In each box plot, the central box shows the average (mean) value, while the horizontal line within the box represents the median. Box plot area represents the interquartile range (IQR). The upper and lower error bar in each box plot represent the maximum and minimum value of each data excluding outliers. Outliers were defined as data points that fall outside the range of 1.5 times the IQR and were represented as black points.
